# Community-based rehabilitation for people with psychosocial disabilities in low- and middle-income countries: a systematic review of the grey literature

**DOI:** 10.1186/s13033-024-00630-0

**Published:** 2024-03-14

**Authors:** Ana-Maria Butura, Grace K. Ryan, Tom Shakespeare, Olusegun Ogunmola, Olubukola Omobowale, Rachel Greenley, Julian Eaton

**Affiliations:** 1 Department of Child & Adolescent Psychiatry, Institute of Psychiatry, Psychology and Neuroscience, Kings College London, 16 De Crespigny Park, London, SE5 8AB UK; 2 Department of Population Health, Centre for Global Mental Health, London School of Hygiene and Tropical Medicine, Keppel Street, London, WC1E 7HT UK; 3 International Centre for Evidence in Disability, Department of Population Health, London School of Hygiene and Tropical Medicine, Keppel Street, London, WC1E 7HT UK; 4 Department of Psychiatry and Centre for Child and Adolescent Mental Health, College of Medicine, University of Ibadan, Ibadan, 200212 Oyo Nigeria; 5 CBM Global Disability Inclusion, Dr.-Werner-Freyberg-Straβe 7, 69514 Laudenbach, Germany

## Abstract

**Background:**

Community based rehabilitation (CBR) aims to promote the inclusion and participation of people with disabilities, particularly in low- and middle-income countries (LMICs). Yet people with psychosocial disabilities are often excluded from CBR programmes. The restrictive inclusion criteria used by previous reviews make it difficult to identify promising examples that could otherwise help to inform the uptake of CBR for people with psychosocial disabilities. We aim to address this gap using gold standard methods for the review and synthesis of grey literature on CBR for people with psychosocial disabilities in LMICs.

**Methods:**

Our search strategy was developed in consultation with an expert advisory group and covered seven grey literature databases, two customised Google Advanced searches, 34 targeted websites and four key reports. A single reviewer screened the search results and extracted relevant data using a standardised format based on the World Health Organisation’s CBR matrix. The included programmes were then checked by a second reviewer with experience in CBR to ensure they met the review’s criteria. A narrative synthesis with summative content analysis was performed to synthesise the findings.

**Results:**

The 23 CBR programmes identified for inclusion spanned 19 countries and were mostly located in either rural areas or urban areas where a large proportion of the population was living in poverty. 13 were classified as livelihood programmes, eight as empowerment programmes, seven as social programmes, seven as health programmes and four as education programmes. Only two addressed all five of these components. 12 of the included programmes reported challenges to implementation, with stigma and lack of resources emerging as two of the most prominent themes.

**Conclusion:**

This grey literature review identified several CBR programmes and synthesised key learning that would have otherwise been missed by a more traditional review of the published literature. However, as evaluation by implementing organisations is not always conducted to a high standard, the quality of this evidence is generally poor. A flexible monitoring and evaluation framework for CBR programmes could help to reduce heterogeneity in terms of the quality and content of reporting.

**Supplementary Information:**

The online version contains supplementary material available at 10.1186/s13033-024-00630-0.

## Background

The Convention on the Rights of Persons with Disabilities (CRPD) [1] highlights the need for rehabilitation services and community participation "to enable people with disabilities to attain and maintain maximum independence, full physical, mental, social and vocational ability” [1, 2]. Community Based Rehabilitation (CBR) is one such approach, which, along with the broader strategy of Community Based Inclusive Development (CBID), has the potential to shift provision of support services from a focus on medical rehabilitation to a human rights perspective [3, 4]. These approaches aim to improve the quality of life of people with disabilities and their families by equalising opportunities, enhancing social inclusion, encouraging independent living, and striving for social justice [3, 5]. Importantly, with greater evidence for the practical application of such approaches, there is the possibility of contributing to deinstitutionalisation.

**Fig. 1:**
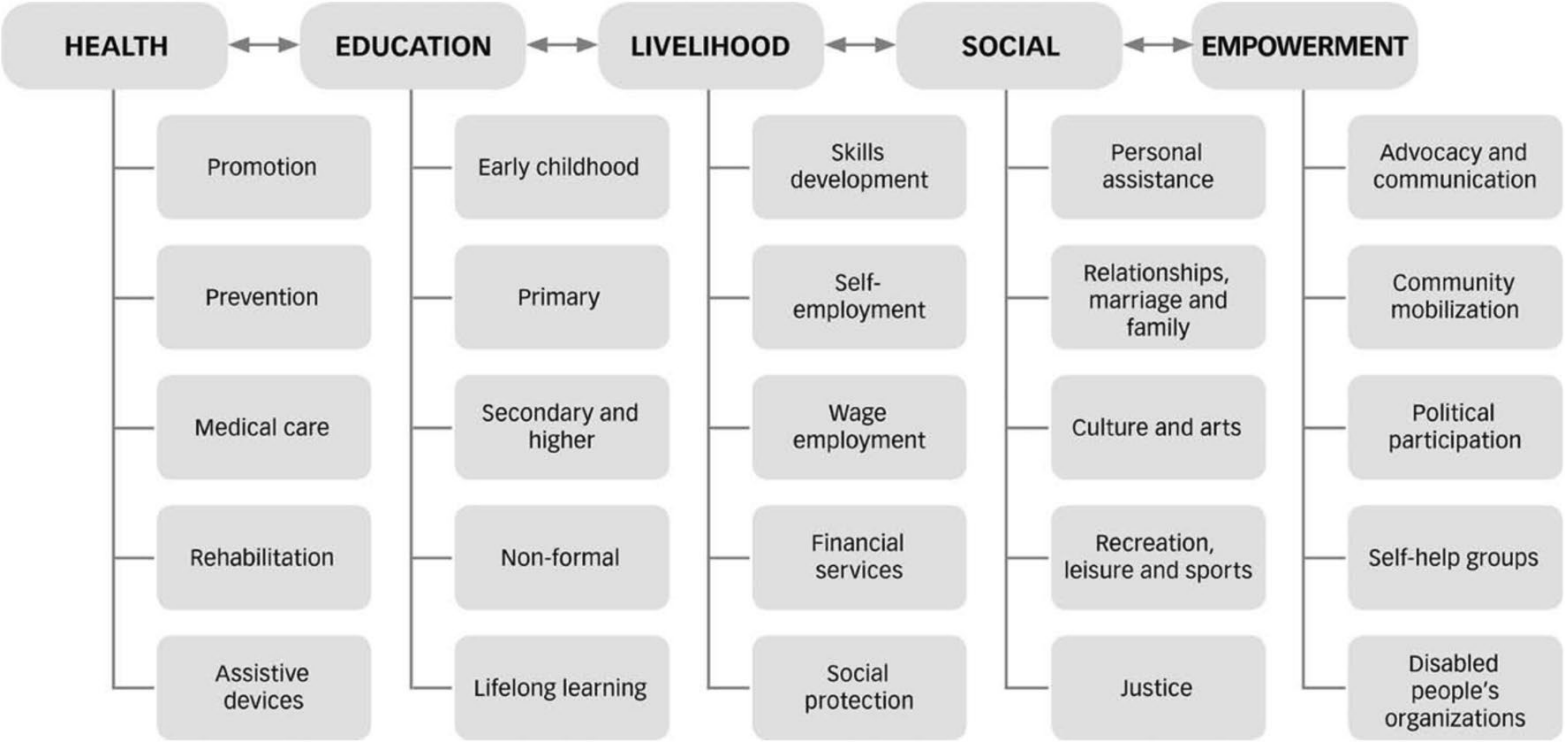
CBR matrix. Source: WHO (2010), Fig. 1, page 25

The CBR matrix integrated into the 2010 CBR guidelines is based on the principles of the CRPD and encourages a multisectoral approach (see Fig. 1) [5]. CBR programmes should include elements across five key components: health, education, livelihood, empowerment, and social [5]. These programmes are delivered in the community, make use of local resources in order to promote sustainability, and are implemented through the combined efforts of people with disabilities, their families, communities, and relevant stakeholders [6].

CBR is intended for all people with disabilities, including those with psychosocial disabilities [7]. However, this group has often been excluded, reflecting a historical neglect of mental health in global health, and of psychosocial disabilities in international development generally [7, 8]. Recognising this, the World Health Organization (WHO), the International Labour Organisation (ILO) and the United Nations Educational, Scientific and Cultural Organization (UNESCO) added a supplementary booklet on mental health to their CBR Guidelines in 2010 [7]. These recommendations drew mainly from expert opinion, evidence in community mental health, and basic development principles. At the time, there were few studies on CBR for people with psychosocial disabilities in low- and middle-income countries (LMICs).

A systematic review conducted by Asher et al. (2017) identified 11 randomised controlled trials for community-based interventions for people with schizophrenia, some of which cover key aspects of the CBR matrix [9]. They reported an overall strong effect on symptom severity, improved functioning, and reduced hospital readmissions [9]. In their systematic review of CBR in LMICs, lemmi et al. (2016) identified nine controlled studies of CBR for people with “mental disability” (schizophrenia, dementia, intellectual disability) and reported that CBR had positive clinical effects for these groups [10]. Both reviews highlighted the limited evidence available from LMICs, and the lack of good-quality trials.

However, these and other similar systematic reviews have only included controlled studies, typically from the peer-reviewed literature, potentially omitting other valuable sources of information. As complex interventions like CBR/CBID are often carried out by civil society, this is a significant oversight. Although one review did include a search of the grey literature, this was limited to only a few sources and did not follow gold standard practices [10]. Given the limited evidence on CBR for people with psychosocial disabilities, it is important to consider all the relevant grey literature [11], particularly when focusing on LMICs, where mental health services are delivered primarily in programmatic settings as opposed to research settings. Understanding the different ways of implementing CBR as a strategy for community development in practice, together with the challenges experienced in programmatic settings, could help to inform the delivery of CBR in LMICs.

### Aims and objectives

This systematic review of the grey literature on CBR for people with psychosocial disabilities in LMICs aims to help inform the integration of mental health into CBR programmes in LMICs. The objectives are:

- To identify similarities and differences among CBR interventions for people with psychosocial disabilities in LMICs;
- To characterise the different ways of implementing these interventions in programmatic settings, as recorded in the grey literature;
- To explore the challenges and experiences of implementation in these settings;
- To appraise the quality of the methods and reporting used in their evaluation.

## Methods

This review applies methods originally developed and piloted by Godin et al. [12] to establish a "gold standard” for systematic reviews of the grey literature [12]. The review protocol was piloted by Ryan (2018) with oversight from an expert advisory panel to identify grey literature on CBR for psychosocial disabilities from three LMICs (Nigeria, Bolivia and Bangladesh) [13]. The protocol was then adapted for this review.

### Eligibility criteria

#### Interventions

Programmes that fit the definition used by Iemmi et al. (2016) were eligible for inclusion [10]. This definition is based on the CBR Guidelines and was originally developed by Lukersmith et al. [14]:

- A programme for people with disabilities and/or their family, their carers, their community;
- Delivered at the community level;
- Implemented through the combined efforts of people with disabilities and/or their family/carer with at least one of the following stakeholder groups: the community, relevant governmental and non-governmental health, education, vocational, social, and other services;
- Focusing on at least two of the following areas: health, education, livelihood, social, empowerment; and
- Forming part of local community development.

Due to the rapidly evolving language in disability-inclusive development, not all programmes that fit the above definition identify as CBR; for example, more recent programmes often refer to themselves as CBID. We, therefore, included programmes with the above characteristics, regardless of whether they used the term “CBR”. Programmes were considered if they included two or more components of the CBR matrix. These could be a mixture of primary (i.e., the main focus of the programme) and minor components.

In keeping with recent systematic reviews of the published literature, programmes delivered primarily in schools or hospitals, clinics, prisons or outpatient centres were not considered to have taken place in community settings and were therefore excluded [9, 10]. However, we did not exclude programmes that offered supplementary services in any of the above settings, recognising that CBR programmes often deliver support across a number of different platforms.

#### Population

Programmes were included if they provided services to people with psychosocial disabilities, following the CRPD definition of disability as outlined above. For the purposes of this review, we operationalised this definition to include people with psychosocial impairments related to psychotic disorders, mood disorders and developmental disorders, based on the categories of the International Classification of Diseases (ICD-10) [17] (See Additional file 1). We did not exclude programmes targeting psychosocial disabilities alongside other disabilities, such as intellectual disabilities, and found that although these groups are very different, many programmes and associated grey literature either do not clearly distinguish between them or wrongly conflate them [15, 16]. Additionally, many countries still use other ambiguous and derogatory language, which contributes to the blurring of these categories. Regardless of the language used by the programme under discussion, this paper uses the term ‘psychosocial disabilities’, so long as the targeted group meets the CRPD definition of psychosocial disability.

#### Setting

All programmes delivered in LMICs based on the World Bank income classification of 2019 [18] were included, as not all texts in the grey literature specify publication dates.

#### Report characteristics

All relevant grey literature was included, such as reports, theses, conference proceedings, newspapers, fact sheets, websites, and policy documents [12]. Where the date of publication could be established, grey literature produced before 1994 was excluded because this is the year the joint WHO, ILO and UNESCO position paper on CBR was published [19]. Only texts in the English language were included; challenges in conducting multi-language searches are a significant limitation even of gold-standard methods for searching the grey literature [12].

#### Study design and outcomes

To be included, texts had to report at least one or more client-, provider- or programme-level outcomes; for example, clients’ functioning, providers’ knowledge, attitudes and practices, or service coverage. There were no other restrictions on study design or comparators. However, texts that only described CBR guidelines were excluded.

### Information sources and search strategies

This review incorporates search strategies from four different sources: grey literature databases; customised Google search; targeted websites; and key reports.

1. The grey literature database search covered the following: PsycExtra (Ovid), Source (International Online Resource Centre on Disability and Inclusion), Global Health (Ovid), UNESCO Library, ILO Library, WHO Library (WHOLIS), Eldis and The Office of the United Nations High Commissioner for Human Rights (OHCHR) Library. (Date searched: 28/06/20–06/08/20)
2. A web search using a customised Google Advanced search engine that was developed for this review. Two separate Google searches were performed for CBR and CBID, respectively. (Date searched: 28/07/20)
3. A hand search of 34 websites of relevant organisations, based on recommendations of an eight-person advisory committee with experience in CBR and CBID and expertise in mental health. (Date searched: 29/07/20–06/08/20)
4. A hand search of four additional reports was recommended by the advisory committee [6, 7, 20, 21]. (Date searched: 20/07/20)

The search strategy (Additional file 2) covered the following three key domains: community-based rehabilitation, psychosocial disabilities and related conditions, and LMICs. The search strategy was developed, piloted, and refined in consultation with a qualified information specialist and informed by several published systematic reviews on related topics [9, 10]. The advisory committee also reviewed the search terms.

### Screening and extraction

The results and dates of each search were exported to an Excel spreadsheet for screening and extraction (Additional file 3). For the web-based searches, the first 100 results from each search were screened, using the title and short description, and bookmarked under the search terms used, which were then manually transcribed into Excel. If the websites did not have a database, a manual page-by-page hand search of the content was then conducted.

The lead author (AB) screened all the titles and descriptions of the texts identified and assessed the full texts against the inclusion criteria. The lead author extracted relevant information into an Excel-based data extraction sheet covering key bibliographic information, setting, programme characteristics, description of services, outcomes, components of CBR matrix adopted, study design, programme outcome and programme challenges. The completed data extraction sheet was then reviewed by two advisors (TS, JE) with experience in CBR to double-check the screening decision based on the programmatic elements described. The full texts were treated as qualitative data and copied into Word documents for hand coding and analysis.

### Quality assessment

A quality assessment for each document was conducted using a modified version of the Authority, Accuracy, Coverage, Objectivity, Date and Significance (AACODS) checklist [22], used by the National Institute for Clinical and Health Excellence (NICE, United Kingdom) as an evidence evaluation checklist for grey literature evaluation [23]. The checklist consists of six domains, which can be answered ‘yes’ if the document meets all the set criteria, ‘partly’ if it meets at least half of the criteria but still differs in others, ‘no’ if it meets less than half of the criteria, ‘unclear’ if there is not enough information in the document to answer, and ‘not applicable’ if the criteria are not relevant. We calculated a total quality score by assigning 2 points to a ‘yes’ answer and 1 point to a ‘partly’ answer in each domain. Each document was scored by at least two reviewers (AB, JE, TS, OO, OO). Inter-rater reliability was calculated in Excel and expressed as the percentage agreement between reviewers.

### Synthesis of results

A narrative synthesis of the data was conducted, following the guidance outlined by Popay et al. [24]. This included a summative content analysis exploring the challenges reported by the programmes. Following an initial phase of data immersion, a logical coding framework that represented the key themes across all the texts was developed through an inductive process of coding by the lead reviewer (AB). The texts were then coded deductively using this initial framework, with emerging themes recorded as memos. The synthesis of results is organised first by the primary CBR components of the programme and then by the challenges reported.

## Results

**Fig. 2:**
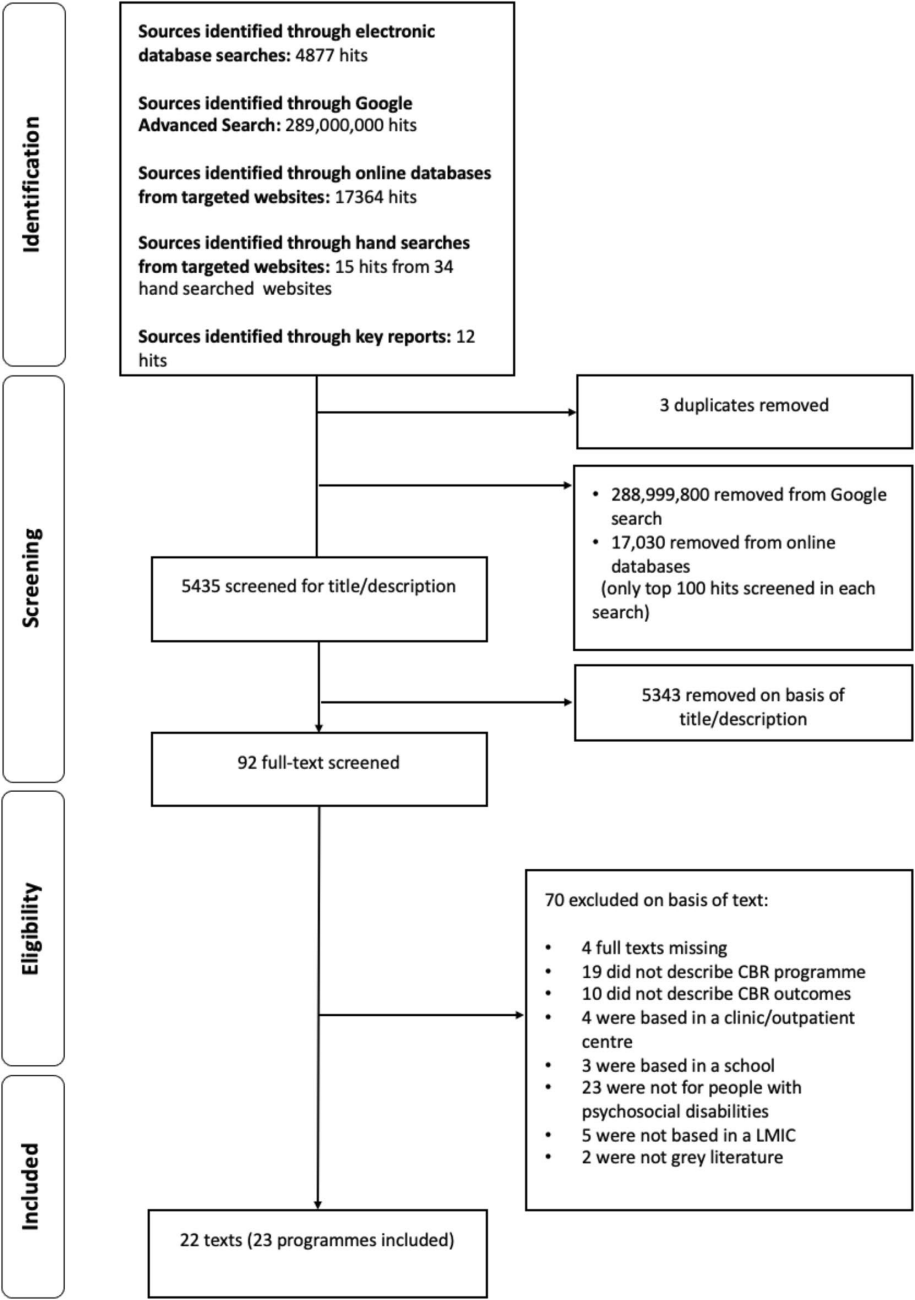
Flow chart of included programmes

The search resulted in 23 programmes being selected for inclusion, as described in 22 texts. The flow diagram for study selection is in Fig. 2. 12 of the programmes came from websites, six from reports, two from good practice guides, two from policy reports and one from an article report (see Table 1 for a summary of the main characteristics of the included programmes.

**Table 1:** Summary characteristics of included studies

Variable		Number of programmes	%
Region	East Asia	2	9
	South Asia	9	39
	Southeast Asia	2	9
	West Asia	1	4
	East Africa	2	9
	West Africa	4	17
	South America	2	9
	Multiple	1	4
Area	Rural	9	39
	Urban	5	22
	Rural and urban slums	1	4
	Not reported	8	35
Type of disability	Psychosocial	13	57
	Intellectual and psychosocial	3	13
	Developmental (Autism)	2	9
	All disabilities, inc. psychosocial or intellectual	5	22
Target group	Adults	21	91
	Children	2	9
Study design	Multi-methods	10	43
	Quantitative	8	35
	Qualitative	5	22

### Quality of documents

Two independent reviewers performed a quality assessment on each of the included texts (one website could no longer be accessed at the time the quality assessment was conducted) [25]. Of these 22 texts, 11 (48%) were rated as having high quality (scoring 9–12) [26–31, 33–35], 7 (30%) were medium quality (scoring 5–8) [36–42], and four (17%) were low quality (scoring 1–4) [43–46]. All of the high-quality documents demonstrated considerable methodological integrity; clearly stated limits; included date; were adjudged as making a significant contribution to knowledge; and all but one had at least a partial score on the authority criterion [28]. The medium-quality literature all satisfied (at least in part) the criteria for accuracy and significance; the inclusion date [27, 36, 37]; were reviewed by a reputable organisation [37, 39–42]; stated limitations [39–42]; and presented findings in an objective manner [36, 37, 39–42]. The low-quality documents did not meet most of the criteria on the checklist. However, some were of considerable authority [45, 46], presented results objectively [43, 44], included date [43, 45], and/or were deemed to have added significant value to the field [43, 44]. Inter-rater agreement scores for each included text ranged from 92 to 100%. (See Additional file 4).

### Description of the included programmes

#### Setting

23 programmes were included, representing 19 LMICs. The majority of programmes took place in Asia, followed by Africa and South America. Although eight programmes did not specify whether they were located in a rural or urban area, most programmes were targeted at people from disadvantaged populations, particularly in rural areas (see Table 1 for the characteristics of each included programme).

#### Population

The majority of programmes were targeted solely at the individual with a disability (n = 15), while the rest were either just for the family (n = 2) or for both the individual and the family (n = 6). Some programmes focused on particular groups of vulnerable people. Four programmes provided services for homeless people living in poverty [4, 27, 33, 43], one of which provided shelter specifically for homeless women at risk of rape and abuse [27]. Another two programmes focused on adults who have either been abandoned [42] or chained and isolated by their family [44].

#### Intervention

**Fig. 3:**
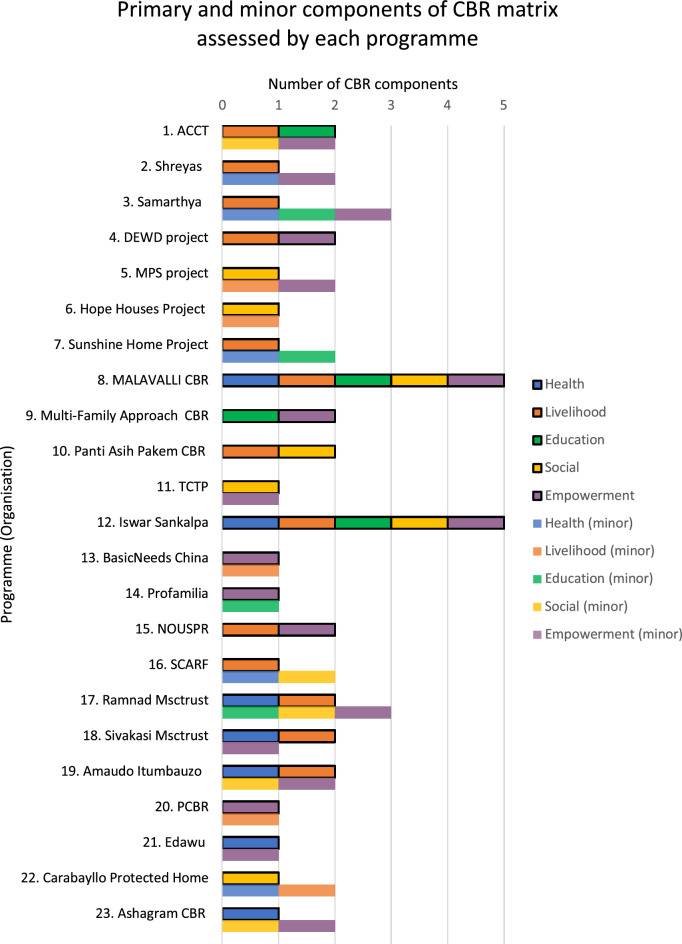
Primary and minor components of CBR matrix assessed by each programme

The search identified programmes under various labels, but only 12 identified themselves as CBR (n = 11) or CBID (n = 1); the remaining 11 were not labelled as such but met the inclusion criteria. 13 of the programmes had one primary CBR component, eight programmes had two primary components, and two of the programmes worked across all the domains of the CBR matrix [27, 38] ‘Livelihood’ and ‘empowerment’ were the primary components for 13 and seven of the programmes, respectively. ‘Social’, ‘health’ and ‘education were also adopted as primary components by seven, seven and four programmes, respectively. Several programmes also included other aspects of the CBR matrix as minor components (see Fig. 3 for a graphic representation of the primary and minor components of the CBR matrix adopted by each programme).

#### Outcomes

Of the 23 programmes, 17 programmes reported impact data, and six reported only implementation outcomes. The impact data suggested improvements in various outcomes such as rehospitalisation rates [31], relapse rates [39], disability [27, 29, 42], quality of life [26, 43, 44], social functioning [44], medication adherence [31, 39], knowledge or awareness of mental condition [25, 27, 28, 37, 39, 40, 44], community reintegration or social inclusion [27, 29, 37, 41–43], employment rates [27, 30, 31, 39, 42, 44], independence [27, 28, 33, 40], stigma [25, 26, 43], governmental support [26, 28, 31], stable housing [31] and homelessness rates [33]. One organisation reported negative outcomes at follow-up before their adoption of a more holistic programme [27]. Many patients discontinued medication, had no support from the family, or were not engaged in community or domestic activities (see Additional file 3 for a detailed description of programme outcomes).

### Synthesis of results

Below we present a synthesis of the review results focusing on the CBR components addressed and challenges to implementation. Please refer to Additional file 3; Data extraction table and summary of results, for details specific to each included programme.

### Components of CBR matrix assessed

#### Livelihood

Livelihood was the most common primary component, focusing mostly on employment. Two programmes in India offer services for people with disabilities, including people with psychosocial disabilities, and conduct awareness-raising activities. The Shreyas Programme trains CBR workers to provide vocational and skills training as well as livelihoods support and counselling [30]. People also have access to employment, technical, material, and financial support services, as well as opportunities to participate in income-generating activities through community banks and self-help groups (SHGs). The Samarthya Programme also offers these services, as well as accommodation, food and transport support, aids and appliances, and therapeutic services [30].

The government-run Sunshine Home Project in China provides community-based day facilities, residential facilities, and home-based visiting services for people with intellectual and psychosocial disabilities [36]. In addition to vocational training, vocational rehabilitation and life skills training, the programme provides medical and psychological services and social and behavioural support. The Schizophrenia Research Foundation in India similarly runs a variety of services for people with schizophrenia, such as clinical services and day centres, including various vocational activities, residential facilities, and social activities [39].

One programme was classified as both livelihood and education. The Autism Awareness Care and Training (AACT) organisation in Ghana [32] offers training for children and parents. Activities include life-skills (bathing, dressing, ironing, etc.), vocational skills (cooking, gardening, etc.), academic education, art, and music therapy. AACT provides children with opportunities to attend mainstream schools to socialise with other children and promote awareness and social inclusion.

Two programmes were classified under the livelihood and empowerment component. The Developing Entrepreneurship among Women with Disabilities (DEWD) project ran in Ethiopia, Kenya, Tanzania, Uganda, and Zambia [35]. Organizations of persons with disabilities (OPDs) supported women with disabilities (including psychosocial disabilities) and women whose children have intellectual disabilities in improving their entrepreneurial skills. A peer support group run by the National Organization of Users and Survivors of Psychiatry (NOUSPR) in Rwanda [46] runs support groups to address individual needs, provide income-generating activities, and train family members to better assist a person with disabilities.

The Panti Asih Pakem CBR Programme in Indonesia consists of both livelihood and social components. It is an intensive two-month training programme for young adults aged 17–25 with psychosocial disabilities [28]. The training has three components including skills for productive work (mechanics, women’s skills, handicrafts, agriculture, cooking, safety in work), healthy lifestyle and recreation, arts, and sports.

Three programmes were classified under the livelihood and health components and all three provided services to homeless people with disabilities. The Ramnad District Mental Health Programme identified people who have been chained and isolated in the district and offered rehabilitation [44]. Community mental health camps provided them with health care, treatment, and entitlements such as a disability certificate. Rehabilitation centres offer vocational training (goat rearing, soap making, etc.) and employment placements to support recovery and reintegration, alongside family psychoeducation, community awareness and self-help groups at the district level. The Sivakasi Rural Community Mental Health Camp provided the same activities but was run by volunteers every month [43]. The Amaudo programme in Nigeria provides medical, occupational, psychological, and social services to homeless people with psychosocial and intellectual disabilities [41]. This programme targets both the service user and their family, with the goal of recovery and community reintegration. Vocational training and employment placements are provided, as well as continued support in their home setting and through self-help groups. Awareness-raising interventions are also carried out in rural communities to reduce stigma and improve early identification.

In general, there was consensus that livelihood was extremely important to service users, but this was a challenging area in the context of general economic hardship in communities. Despite this, good outcomes were reported, though traditional ‘vocational training’ was not considered sufficient. There was usually a need for more intensive interventions, including support, once people were in work.

#### Empowerment

Self-help groups (SHGs) are a central means of facilitating empowerment, for example, in the Presbyterian Community Based Rehabilitation programme in Ghana [40], people with psychosocial disabilities take an active role in the decision-making and organisation of the groups. In the Liang Fen Zhuang SHGs in China, supported by BasicNeeds [25], the group plants and sells grapes in the community charity bazaar. This allows them to gain an income but also served to raise awareness of mental health. Additionally, the Profamilia organisation, along with other organisations in Colombia, addresses the sexual and reproductive rights of people with intellectual and psychosocial disabilities [34]. They developed a training tool that supports people in making decisions on these issues for persons with disabilities and their families, and for judges and health professionals.

One programme comprised both empowerment and education components. The Multi-Family Approach brings together mothers of children with a mental or physical disability in Palestine, West Bank [26] to share experiences and learn from each other.

#### Health

Health was linked to many programmes, either delivering care ‘in-house’ or linked to a referral service. The Ashagram CBR programme in India provides a range of services for people with schizophrenia [29]. As well as a mental health clinic, they have trained local community members as CBR workers to provide comprehensive home-based services. The Edawu Community Mental Health Care Project in Nigeria provides inpatient medical care and rehabilitation for homeless people with follow-up after discharge [33]. The programme also aims to improve the early identification of mental illness by delivering mhGAP training through staff workshops, role-playing activities and lectures. Both programmes also developed a community mental health awareness programme to combat stigma.

#### Social

Programmes identified under the social component provided stable, independent housing for people with psychosocial disabilities to encourage a move away from institutional care. The Mental Health Policy and Service Development project in Sri Lanka relocates people who were previously institutionalised and provides them with stable housing or integrates them back into family homes [31]. The programme also provides them with vocational training and employment support and raises awareness about mental health in the community.

Hope Houses in Turkey is a project that provides accommodation to a maximum of six disabled people in houses under caretaker supervision [45]. The project aims to encourage independent living in their local community by providing education, employment and psychosocial support and encouraging community connections through relationships with their neighbours as well to make their own choices in daily life, shopping, recreation, and work. In Peru (Carabayllo), the Protected Home programme provides housing and rehabilitation care for women with severe mental conditions who have been abandoned [42]. Community health agents provide self-care workshops, individual and group therapies and social skills for community integration and autonomy.

A sports training programme in Thailand and Japan with various participants from Southeast Asia aimed to facilitate inclusive participation of people with autism and psychosocial disabilities in national and international competitions [37]. Potential athletes and their parents were trained and participated in empowering games, activities, and competitions.

#### Livelihood, empowerment, health, social and education

Two large organisations in India worked under all five CBR components. The Iswar Sankalpa organisation in India supports homeless people with psychosocial disabilities [27] with several programmes that aim to prevent homelessness and provide medical and social treatment and support, shelter for women and men, day care centres, livelihood activities, education centres and assisted community living. The Shree Ramana Maharishi Academy organises the Malavalli CBR programme for the Blind in India [38]. It provides people with psychosocial disabilities health and medical rehabilitation, education, livelihood support, leadership training for SHGs and OPDs, and social activities.

Figure 3 shows the number of primary (first bar) and minor (second bar) components each programme adopted. Each unit represents one component. The programmes are numbered in the same order as per the data extraction table (see Additional file 3).

#### Challenges to implementation

**Fig. 4:**
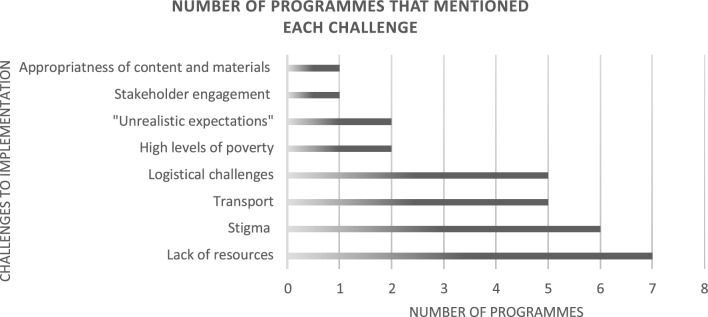
Number of programmes mentioning specific challenges to implementation

12 out of the 23 included programmes reported challenges related to implementation. Eight key themes emerged from these texts. See Fig. 4 for a graphic representation of the number of programmes that mentioned each challenge. (See also Additional file 5 for a detailed breakdown of challenges).

#### Limited/lack of resources

The challenge most commonly reported was the limited or lack of available resources. The Ashagram and Presbyterian CBR programmes reported a lack of financial and human resources generally [29–40]. Five programmes reported more specific challenges regarding human resources: the Mental Health Policy and Service Development project provided different services in “two districts due to shortages of human resources” [31]; the Protected Home programme reported “difficulty recruiting appropriate community health workers for the recruitment of caregivers” [42]; the Edawu programme found it difficult to source medicines in rural areas [33] and reported that due to the “remote location of the project” it is difficult to “attract qualified staff who may be used to urban lifestyles”; The Panti Asih Pakem programme reported “a lack of human resources for programme development and management”, as “people demand free in-house rehabilitation” [28]; and The Multifamily approach in Palestine [26] “struggled to provide support for…mothers with severely disabled children…who need special care”, and the volunteers did not have appropriate training to look after them.

#### Stigma

The second most common challenge was stigma from the community or family members. Stigma was described in two ways: as prejudice or discrimination. Prejudice was often described as “negative attitudes”, such as: attitudes towards women with mental health difficulties in the workforce, leading to their exclusion [30, 35]; towards those seeking mental health support [26, 40]; and towards employment of women graduates [30]. Discrimination was also mentioned by the Amaudo programme who described “high levels of stigmatisation and discrimination of people with mental health under rehabilitation by family and society which resulted in poor care at home and high risk of relapse” [41].

#### Transport

As most programmes were based in rural areas, accessible transport was a significant barrier highlighted by five programmes. This was because of either: “poor accessibility to many sections of the catchment area” [29]; individual financial limitations [30, 31]; or individuals “working from long distances… [that made it] difficult for them to attend sessions” [26]; or no access to the only mode of transport due to a government policy [41]. One programme overcame this challenge by “establishing free transport services to encourage as many people to attend meetings and training [as possible]” [31].

#### Logistical challenges

Logistical challenges to implementation included “poor provision of social infrastructures like roads and electricity… adding burden and costs to travel” [41]; lack of public health infrastructure [29, 31]; and limited access to computers with “fragile and unpredictable internet” [33]. One programme noted that local and more widespread political conflict in Nigeria “makes it difficult for foreign partners to visit or travel freely” [41].

#### High levels of poverty

High levels of poverty were reported as a challenge by two programmes [29, 40]. The Presbyterian Community Based Rehabilitation in Ghana programme reported that the high level of poverty was a barrier to joining self-help groups because they require membership fees [40]. Ashagram in India reported that over 80% of the catchment area were living in poverty and that this was a challenging social demographic; however, they did not elaborate on exactly what challenges this evoked.

#### “Unrealistic expectations”

Two programmes report clients as having “unrealistic expectations” of the CBR programme. They both described these as expectations beyond mental health, such as “financial gain” [40] or “income-generating initiatives and medicines for their disabled children” [26]. The programmes reported having to carefully manage expectations and discuss them with the participants to maintain their commitment. This suggests that there is a high level of need that perhaps programmes are not resourced to meet.

#### Stakeholder engagement

Stakeholder engagement was reported as a challenge by the Amaudo programme which described difficulties in designing and implementing effective policies for mental health care due to unsuccessful attempts at engaging with government officials and policymakers [41].

#### Appropriateness of training content and materials

The Samuha Samarthya programme in India reported difficulty in making the content suited to the participants; certain vocational training materials and content made it difficult for some people with disabilities to participate due to their “limited English language proficiency and computer literacy” [30].

## Discussion

### Summary of findings and implications

23 CBR programmes met the eligibility criteria and were included in a narrative synthesis focused on identifying common elements of the CBR matrix and challenges reported. Although 19 LMICs were represented in this review, countries from the WHO’s Eastern Mediterranean, Europe and the Americas regions were under-represented—likely due to this review’s language restrictions. Furthermore, while CBR is intended for both children and adults, only two programmes focus on children. This finding is consistent with the 2016 systematic review of CBR, which found only one study focusing on children [10].

Although most of the programmes indicated that they were designed for people with psychosocial disabilities, intellectual and developmental disabilities are often reported together with psychosocial disabilities, making it difficult to address the level of inclusion of people in these different groups [16, 47]. A recent review on mental health and psychosocial disabilities in CRPD Country Reports found that many countries did not distinguish between psychosocial and intellectual disabilities and used other ambiguous and derogatory language [15]. This may be due to the lack of resources for diagnosis in LMICs, adopting a different classification paradigm (e.g., disability framework focused on social barriers as opposed to impairments) or explanatory model (e.g., spiritual causes), lack of knowledge and awareness, or translation issues in countries where English is not the national language [8, 47, 48]. Programmes may benefit from culturally specific information and guidelines to improve reporting [15].

A surprising finding is that only six of the programmes included the family in the rehabilitation process. Yet family involvement is essential, particularly in LMICs where formal social safety nets are weak, and is especially important for effective deinstitutionalisation [8, 49] Stigma and fear of discrimination by the community can discourage carers [50]. Positive indicators of caregiving (lower subjective burden and perceived environmental impact, higher caregiving mastery and satisfaction) have been linked to better social functioning, higher employment rates and shorter duration of illness in service users, as well as improved caregiver satisfaction and gratification [51, 52]. Caregivers should be encouraged to join self-help or support groups to increase their confidence and knowledge on how to best support their relatives [51, 53].

Another interesting finding is the relatively large proportion of programmes identified by this review that are not health-focused, compared to those identified by previous reviews [9, 10]. This may reflect key differences in the “real world” grey literature versus the research literature on CBR in LMICs. Asher et al. (2017) posit that given the minimal resources available for mental health in many LMICs, it may be possible to make a measurable difference in patient outcomes even with a fairly narrow focus on health; however, the influence of poverty requires drawing on community resources and addressing livelihood issues [9]. Habtamu et al. [54] suggest that within rural communities, maintaining self-care, productivity in work, engaging in family life and fulfilling social obligations are the most highly valued domains of functioning for people with psychosocial disabilities [54]. This highlights the importance of non-health components which are often underrepresented in the evidence base. Though it is also possible that the focus on health is an artefact of the databases selected for previous reviews (e.g., PubMed/Medline) and/or the relative ease of measuring health outcomes using the comparative study designs included in these reviews—compared to other outcomes that might be more valued by beneficiaries themselves.

Greater focus on social and empowerment components may also reflect programmes’ emphasis on participation. Out of the 23 included programmes, seven were run by people with psychosocial disabilities (or by their families) or involved strong partnerships with OPDs, and 12 supported SHGs. This is in keeping with the principles of “nothing about us, without us”, involving people with disabilities in the design and development of programmes and respecting their right to make their own decisions [55, 56]. Effective CBR programmes should engage people with disabilities in all aspects of their treatment and rehabilitation, and the role of a professional or facilitator should be that of an ally and “co-learner” rather than monopolising the process [57, 59]. This can shift priorities from medical rehabilitation toward employment, education, and poverty alleviation, taking a more consumer-focused approach [59].

This review reinforces the importance of including several CBR components within programmes, but we found surprisingly few did this [5, 60]. The Iswar Sankalpa organisation reported no community engagement, no medicine adherence, and high levels of relapse in their clients at follow-up when focusing solely on medical rehabilitation. The programme saw improvements in their clients only after they “developed longer engagement with patient and family, introduced a deeper assessment of the family needs and community resources and developed community help networks such as SHGs, and vocational opportunities” [27].

Stigma and a lack of resources were two of the most prominent challenges. This is in line with Brooke-Sumner et al.’s [61] systematic review of the feasibility and acceptability of psychosocial interventions for people with schizophrenia in LMICs. They reported stigma as a significant barrier and emphasised the need to support those experiencing stigma to minimise its negative impact [61]. Community-based awareness and advocacy campaigns, the use of mass media and activities that encourage social contact have been identified as crucial for the realisation of CBR principles, [53, 62, 63], and it was encouraging to see that several programmes were working to change community attitudes. Article 19 of CRPD (the right to live independently and be included in the community) is more likely to be realised where both structural and attitudinal issues are addressed. (1(p.13)).

13 of the included studies reported conducting awareness-raising campaigns, but there is a need for more evidence-based approaches to achieve impact. A community-based mental health awareness programme in Nigeria found that increasing community awareness increased demand and referrals to CBR services, as well as local political involvement [62]. However, research on the factors influencing political will among different policymakers is needed to inform such advocacy activities and increase their likelihood of success [64]. The well-established finding that increasing social contact between the community and persons with mental disorders is the most effective intervention for reducing stigma and discrimination [63] further supports the need for livelihood, social and empowerment aspects of CBR that engage participants in all aspects of community life.

The unwillingness of mental health professionals to live and work in rural areas with poor infrastructure exacerbates human resource limitations in LMICs [65]. Meanwhile, low spending on mental health in LMICs by governments and charitable organisations constrains programme development [65] and is particularly damaging in poorer, rural areas where needs are also higher [66, 67]. Greater stakeholder engagement is crucial to make mental health a priority on the political agenda, as well as developing the community workforce with appropriate skills to increase the availability of human resources [53, 65, 68].

### Strengths and limitations

This grey literature review used rigorous, systematic methods to shed light on how CBR programmes operate in practice outside the context of highly controlled research studies. However, grey literature is particularly subject to bias, as reports are typically published by the same organisations responsible for programmes and without transparent processes for peer review. The level of detail and type of findings reported were inconsistent, with some texts reporting impact data and some only reporting implementation data. Although the aim of this grey literature review was not primarily to evaluate impact, a common framework for reporting programmes would be beneficial for this developing field [12, 69] to identify what appears to be effective or ineffective in ‘real world’ practice.

Additionally, as this is a grey literature review essentially concerned with identifying common features of programmes in practice, there is substantial heterogeneity both in terms of the types of programmes included and the amount of detail provided, which makes it difficult to present a critical analysis. Future research should consider how programmes align (or fail to align) with key articles of the UN CRPD, the WHO’s good practice guidelines on community mental health [70], and other frameworks promoting rights-based approaches for people with psychosocial disabilities. For example, several programmes [27, 39, 42, 45] incorporate a residential component, which may be intended as a short-term solution to help people in extremely vulnerable situations transition out of homelessness or neglectful or abusive family arrangements. However, residential care can all too easily equate to institutionalisation, in violation of Article 19 of the CRPD. This further underscores the importance of our call for more systematic documentation and evaluation of CBR/CBID programmes in LMICs, with special attention to potential harms.

Additional methodological limitations of this review include the exclusion of reports not published in English which may have impacted the breadth and variety of programs represented, and not independently double-coding full texts—which was not possible due to resource limitations. Web-based searches are especially difficult to replicate and present special challenges when double-screening [12]. Additional grey literature searches should be conducted in other languages to harness learning from programmes in non-Anglophone countries. Reliability could be improved if results were fed back to topical experts, authors of included texts and representatives of relevant organisations, with a request to identify additional texts that searches may have missed and provide further information about programmes.

## Conclusions

In programmatic settings, rehabilitation services foster empowerment and self-determination by focusing primarily on promoting economic and social inclusion. Reports by these programmes highlight the impact of other non-health aspects of CBR and complement recent reviews of the effectiveness of CBR for people living with psychosocial and intellectual disabilities in LMICs. CBR has the potential to make a significant difference in the lives of people with psychosocial and intellectual disabilities in LMICs. However, most CBR is done outside of research settings, and evaluation is often weak. On the other hand, research organisations tend to favour what can be easily measured rather than complex and diverse social interventions and outcomes. As a result, evidence for the effectiveness and potential impact of CBR is often fragmented and insufficient [69]. There is value in engaging with grey literature, which can broaden the scope of findings captured by the more formal research literature [11].

This review emphasises the need for a CBR monitoring and evaluation framework to encourage programmes to systematically document their work and enable more meaningful comparisons to be made between different programmes. Evidence suggests that a “one-size-fits-all framework” is not appropriate for CBR and that a variety of formal and informal strategies need to be considered to develop a flexible monitoring and evaluation tool that considers resource implications [14, 69, 71]. Additionally, given the ongoing debate about terminology related to CBR, more research is needed to clearly define the key elements required for quality CBR monitoring and evaluation. These can ultimately be an instrument and catalyst for the integration and prioritisation of mental health into CBR and wider development sectors. This is particularly crucial for an area where institutionalisation remains a major feature of services, and a lack of community alternatives is often cited as a barrier to change by decision makers [72].

### Supplementary Information

**Additional file 1: **Categories of the International Classification of Diseases (ICD-10).

**Additional file 2: **Search terms.

**Additional file 3: **Data extraction table; summary of results.

**Additional file 4: **Quality assessment scoring and Inter-rater score.

**Additional file 5: **Additional extraction: Reported challenges to implementation.

## Data Availability

All data generated or analysed during this study are included in this published article and its additional files.

## Abbreviations

CBR: Community based rehabilitation
CBID: Community based inclusive development
LMICs: Low- and middle-income countries
CRPD: Convention on the Rights of Persons with Disabilities
WHO: World Health Organization
ILO: International Labour Office
UNESCO: United Nations Educational, Scientific and Cultural Organization
OHCHR: The Office of the United Nations High Commissioner for Human Rights
OPD: Organizations of persons with disabilities
NGO: Non-governmental organisation
SHGs: Self-help groups
