# Supplementary material for: Community-based rehabilitation for people with psychosocial disabilities in low- and middle-income countries: a systematic review of the grey literature
Source: Int J Ment Health Syst. 2024 Mar 14;18:13. doi: 10.1186/s13033-024-00630-0 (PMC10941461; doi:10.1186/s13033-024-00630-0)
Supplement: Supplementary file 1 — Additional file 1: Categories of the International Classification of Diseases (ICD-10). [file 13033_2024_630_MOESM1_ESM.docx]

**Additional File 1: Categories of the International Classification of Diseases (ICD-10)**

• Organic, including symptomatic, mental disorders (F00-F99)

• Schizophrenia, schizotypal, and delusional disorders (F20-F29)

• Mental retardation (F70-79)

• Disorders of psychological development (F80-F89)

• Mood (affective) disorders:

- F31: Bipolar affective disorder.
