# Supplementary material for: Community-based rehabilitation for people with psychosocial disabilities in low- and middle-income countries: a systematic review of the grey literature
Source: Int J Ment Health Syst. 2024 Mar 14;18:13. doi: 10.1186/s13033-024-00630-0 (PMC10941461; doi:10.1186/s13033-024-00630-0)
Supplement: Supplementary file 2 — Additional file 2: Search terms. [file 13033_2024_630_MOESM2_ESM.docx]

## Additional File 2: Search terms

## Electronic databases

**PsycEXTRA (OvidSP)** 1908 to 2020 – Date searched: 06/09/20

*Adapted from Iemmi et al. (2015)

1. (Community-based rehabilitation or Community based rehabilitation or CBR or Community-based inclusive development or community based inclusive development or CBID or community based mental health programme or community mental health or community treatment).sh,ti,ab.

2. (Communit* adj5 (rehabilitat* or health care or healthcare or health service* or health nursing* or health visitor* or health network* or care network* or counsel* or foster home* or foster care* or home care* or homecare or domiciliary care* or preventive health or health education or health promotion or self-help device* or assistive device*)).sh,ti,ab.

3. (Communit* adj5 inclusi* adj5 (education or school* or preschool* or high-school* or environment* or curricul*)).sh,ti,ab.

4. (Communit* adj5 (vocational training or apprenticeship* or employment placement service* or support network* or self-employ* or social service* or social work*)).sh,ti,ab.

5. (Communit* adj5 (personal assistance or personal assistant* or individual support* or disabled people* organization* or disabled person* organisation*)).sh,ti,ab.

6. (Communit* adj5 (empower* or awareness campaign* or self-advocacy or self-help group* or support group* or women group* or political group* or development group*)).sh,ti,ab.

7. (Communit* adj5 inclusi* adj5 (health or education or hous* or social or justice or empower*)).sh,ti,ab.

8. (rehabilitat* adj5 (home based or home-based)).sh,ti,ab.

9. (exp rehabilitation/ or ((exp health care services/ or exp social services/ or exp community development/) and rehabilitat*.sh,ti,ab.)) and communit*.sh,ti,ab.

10. exp Home Care/ and rehabilitat*.sh,ti,ab.

11. 1 or 2 or 3 or 4 or 5 or 6 or 7 or 8 or 9 or 10

12. (Schizophreni* or Psychos* or Psychotic Disorder* or Schizoaffective Disorder* or Schizophreniform Disorder* or Dementia* or Alzheimer* or Bipolar* or Hypomania or Mania or Manic).sh,ti,ab.

13. exp schizophrenia/ or exp psychosis/ or exp dementia/ or exp Alzheimers Disease/ or exp bipolar disorder/

14. ((Intellectual* or Mental* or Psychological* or Developmental or Psychosocial) adj5 (impair* or retard* or deficienc* or disable* or disabili* or handicap* or ill* or disorder*)).sh,ti,ab.

15. ((communication or language or speech or learning) adj5 disorder*).sh,ti,ab.

16. (Autis* or Dyslexi* or Down* Syndrome or Mongolism or Trisomy 21).sh,ti,ab.

17. exp mental retardation/ or exp developmental disabilities/ or exp pervasive developmental disorders/

18. 12 or 13 or 14 or 15 or 16 or 17

19. (Afghanistan or Albania or Algeria or American Samoa or Angola or Argentina or Armenia or Azerbaijan or Bangladesh or Belarus or Byelarus or Byelorussia or Belorussia or Belize or Benin or Bhutan or Bolivia or Bosnia or Herzegovina or Hercegovina or Bosnia-Herzegovina or Bosnia-Hercegovina or Botswana or Brazil or Brasil or Bulgaria or Burkina Faso or Upper Volta or Burundi or Urundi or Cambodia or Republic of Kampuchea or Cameroon or Cameroons or Cape Verde or Cabo Verde or Central African Republic or Chad or China or Colombia or Comoros or Comoro Islands or Comores or Congo or DRC or Zaire or Costa Rica or Cote dIvoire or Ivory Coast or Cuba or Djibouti or Obock or French Somaliland or Dominica or Dominican Republic or Ecuador or Egypt or United Arab Republic or El Salvador or Eritrea or Ethiopia or Eswatini or Fiji or Gabon or Gabonese Republic or Gambia or Georgia or Ghana or Gold Coast or Grenada or Guatemala or Guinea or Guinea-Bissau or Guiana or Guyana or Haiti or Honduras or India or Indonesia or Iran or Iraq or Jamaica or Jordan or Kazakhstan or Kenya or Kiribati or North Korea or Democratic Peoples Republic of Korea or DPRK or Kosovo or Kyrgyzstan or Kirghizstan or Kirgizstan or Kirghizia or Kirgizia or Kyrgyz or Kirghiz or Kyrgyz Republic or Lao or Laos or Lebanon or Lesotho or Basutoland or Liberia or Libya or Macedonia or Madagascar or Malagasy Republic or Malawi or Nyasaland or Malaysia or Malaya or Malay or Maldives or Mali or Marshall Islands or Mauritania or Mayotte or Mexico or Micronesia or Moldova or Moldovia or Mongolia or Montenegro or Morocco or Mozambique or Myanmar or Burma or Namibia or Nepal or Nicaragua or Niger or Nigeria or Pakistan or Palestine or Papua New Guinea or Paraguay or Peru or Philippines or Russia or Russian Federation or USSR or Soviet Union or Union of Soviet Socialist Republics or Rwanda or Ruanda-Urundi or Samoa or Solomon Islands or Sao Tome or Principe or Senegal or Serbia or Montenegro or Yugoslavia or Sierra Leone or Solomon Islands or Somalia or South Africa or Sri Lanka or Ceylon or Saint Christopher Island or Saint Lucia or St Lucia or Saint Vincent or St Vincent or Grenadines or Sudan or South Sudan or Suriname or Surinam or Swaziland or Syria or Syrian Arab Republic or Tajikistan or Tadzhikistan or Tadjikistan or Tanzania or Thailand or Timor-Leste or East Timor or Togo or Togolese Republic or Tonga or Tunisia or Turkey or Turkmenistan or Turkmenia or Tuvalu or Uganda or Ukraine or Uzbekistan or Vanuatu or New Hebrides or Venezuela or Vietnam or Viet Nam or West Bank or Gaza or Yemen or Zambia or Zimbabwe or Rhodesia).sh,ti,ab.

20. (Africa or Asia or Caribbean or West Indies or Latin America or Central America or South America or Middle East or Eastern Europe or East Europe).sh,ti,ab.

21. ((Developing or Low-income or low income or lower income or Middle-income or Middle income or (Low and middle income) or (Low- and middle-income) or Less-Developed or Less Developed or Least Developed or Under Developed or underdeveloped or Third-World or poor or poorer) adj5 (countr* or nation* or world or econom* or population*)).sh,ti,ab.

22. (LIC or LICs or MIC or MICs or LMIC or LMICs or LAMIC or LAMICs or LAMI countr* or third world).sh,ti,ab.

23. (Transitional countr* or Transitional econom* or Transition countr* or Transition econom*).sh,ti,ab.

24. exp Developing Countries/

25. 19 or 20 or 21 or 22 or 23 or 24

26. 11 and 18 and 25

27. limit 26 to (English language and yr=“1994 –Current”)

**Global Health (OvidSP)** 1910 to 2020 – Date searched: 31/07/20

1. (Community-based rehabilitation or Community based rehabilitation or CBR or Community-based inclusive development or community based inclusive development or CBID or community based mental health programme or community mental health or community treatment).sh,ti,ab.

2. (Communit* adj5 (rehabilitat* or health care or healthcare or health service* or health nursing* or health visitor* or health network* or care network* or counsel* or foster home* or foster care* or home care* or homecare or domiciliary care* or preventive health or health education or health promotion or self-help device* or assistive device*)).sh,ti,ab.

3. (Communit* adj5 inclusi* adj5 (education or school* or preschool* or high-school* or environment* or curricul*)).sh,ti,ab.

4. (Communit* adj5 (vocational training or apprenticeship* or employment placement service* or support network* or self-employ* or social service* or social work*)).sh,ti,ab.

5. (Communit* adj5 (personal assistance or personal assistant* or individual support* or disabled people* organization* or disabled person* organisation*)).sh,ti,ab.

6. (Communit* adj5 (empower* or awareness campaign* or self-advocacy or self-help group* or support group* or women group* or political group* or development group*)).sh,ti,ab.

7. (Communit* adj5 inclusi* adj5 (health or education or hous* or social or justice or empower*)).sh,ti,ab.

8. (rehabilitat* adj5 (home based or home-based)).sh,ti,ab.

9. (exp rehabilitation/ or ((exp health care services/ or exp social services/ or exp community development/) and rehabilitat*.sh,ti,ab.)) and communit*.sh,ti,ab.

10. exp Home Care/ and rehabilitat*.sh,ti,ab.

11. 1 or 2 or 3 or 4 or 5 or 6 or 7 or 8 or 9 or 10

12. (Schizophreni* or Psychos* or Psychotic Disorder* or Schizoaffective Disorder* or Schizophreniform Disorder* or Dementia* or Alzheimer* or Bipolar* or Hypomania or Mania or Manic).sh,ti,ab.

13. exp schizophrenia/ or exp psychosis/ or exp dementia/ or exp Alzheimers Disease/ or exp bipolar disorder/

14. ((Intellectual* or Mental* or Psychological* or Developmental or Psychosocial) adj5 (impair* or retard* or deficienc* or disable* or disabili* or handicap* or ill* or disorder*)).sh,ti,ab.

15. ((communication or language or speech or learning) adj5 disorder*).sh,ti,ab.

16. (Autis* or Dyslexi* or Down* Syndrome or Mongolism or Trisomy 21).sh,ti,ab.

17. exp mental retardation/ or exp developmental disabilities/ or exp pervasive developmental disorders/

18. 12 or 13 or 14 or 15 or 16 or 17

19. (Afghanistan or Albania or Algeria or American Samoa or Angola or Argentina or Armenia or Azerbaijan or Bangladesh or Belarus or Byelarus or Byelorussia or Belorussia or Belize or Benin or Bhutan or Bolivia or Bosnia or Herzegovina or Hercegovina or Bosnia-Herzegovina or Bosnia-Hercegovina or Botswana or Brazil or Brasil or Bulgaria or Burkina Faso or Upper Volta or Burundi or Urundi or Cambodia or Republic of Kampuchea or Cameroon or Cameroons or Cape Verde or Cabo Verde or Central African Republic or Chad or China or Colombia or Comoros or Comoro Islands or Comores or Congo or DRC or Zaire or Costa Rica or Cote dIvoire or Ivory Coast or Cuba or Djibouti or Obock or French Somaliland or Dominica or Dominican Republic or Ecuador or Egypt or United Arab Republic or El Salvador or Eritrea or Ethiopia or Eswatini or Fiji or Gabon or Gabonese Republic or Gambia or Georgia or Ghana or Gold Coast or Grenada or Guatemala or Guinea or Guinea-Bissau or Guiana or Guyana or Haiti or Honduras or India or Indonesia or Iran or Iraq or Jamaica or Jordan or Kazakhstan or Kenya or Kiribati or North Korea or Democratic Peoples Republic of Korea or DPRK or Kosovo or Kyrgyzstan or Kirghizstan or Kirgizstan or Kirghizia or Kirgizia or Kyrgyz or Kirghiz or Kyrgyz Republic or Lao or Laos or Lebanon or Lesotho or Basutoland or Liberia or Libya or Macedonia or Madagascar or Malagasy Republic or Malawi or Nyasaland or Malaysia or Malaya or Malay or Maldives or Mali or Marshall Islands or Mauritania or Mayotte or Mexico or Micronesia or Moldova or Moldovia or Mongolia or Montenegro or Morocco or Mozambique or Myanmar or Burma or Namibia or Nepal or Nicaragua or Niger or Nigeria or Pakistan or Palestine or Papua New Guinea or Paraguay or Peru or Philippines or Russia or Russian Federation or USSR or Soviet Union or Union of Soviet Socialist Republics or Rwanda or Ruanda-Urundi or Samoa or Solomon Islands or Sao Tome or Principe or Senegal or Serbia or Montenegro or Yugoslavia or Sierra Leone or Solomon Islands or Somalia or South Africa or Sri Lanka or Ceylon or Saint Christopher Island or Saint Lucia or St Lucia or Saint Vincent or St Vincent or Grenadines or Sudan or South Sudan or Suriname or Surinam or Swaziland or Syria or Syrian Arab Republic or Tajikistan or Tadzhikistan or Tadjikistan or Tanzania or Thailand or Timor-Leste or East Timor or Togo or Togolese Republic or Tonga or Tunisia or Turkey or Turkmenistan or Turkmenia or Tuvalu or Uganda or Ukraine or Uzbekistan or Vanuatu or New Hebrides or Venezuela or Vietnam or Viet Nam or West Bank or Gaza or Yemen or Zambia or Zimbabwe or Rhodesia).sh,ti,ab.

20. (Africa or Asia or Caribbean or West Indies or Latin America or Central America or South America or Middle East or Eastern Europe or East Europe).sh,ti,ab.

21. ((Developing or Low-income or low income or lower income or Middle-income or Middle income or (Low and middle income) or (Low- and middle-income) or Less-Developed or Less Developed or Least Developed or Under Developed or underdeveloped or Third-World or poor or poorer) adj5 (countr* or nation* or world or econom* or population*)).sh,ti,ab.

22. (LIC or LICs or MIC or MICs or LMIC or LMICs or LAMIC or LAMICs or LAMI countr* or third world).sh,ti,ab.

23. (Transitional countr* or Transitional econom* or Transition countr* or Transition econom*).sh,ti,ab.

24. exp Developing Countries/

25. 19 or 20 or 21 or 22 or 23 or 24

26. 11 and 18 and 25

27. limit 26 to (English language and yr=“1994 –Current”)

28. limit 27 to (annual report or annual report section or bulletin or bulletin article or conference or conference paper or conference proceedings or correspondence or editorial or patent or standard or miscellaneous or thesis)

**Other Electronic Databases**

**ILO –** Date searched: 20/07/20

- Search [Free text]: Community based rehabilitation mental
- Language: English
- Topic: Developing Countries

**UNESDOC –** Date searched: 17/07/20

- Search [Free text]: Community based rehabilitation mental
- Language: English
- Exclude years of publication before 1994

**WHOLIS –** Date searched: 21/07/20

Advanced search:

- With all of these words: community based rehabilitation
- With at least one of these words: mental psychosocial
- Language: English
- 20 results per page
- Topic: Mental disorder

**UNITED NATIONS HUMAN RIGHTS LIBRARY –** Date searched: 28/06/20

- Search Free text): (''community based rehabilitation'' OR ''community based inclusive development'') AND (''psychosocial disabilities'' OR ''mental'')

**ELDIS -**  Date searched: 28/07/20

- Search [Free text]: Community based rehabilitation mental
- Type: Documents or News, Blogs & Key Issues
- Published between 1995-2020

**SOURCE –** Date searched: 04/07/20

- Search Resources [Free text]: Community based rehabilitation mental
- Select Language: English

**Google Advanced Search –** Date searched: 28/07/20

**Google Settings**

- Turn off Web and App Activity
- Turn off Location History
- Turn off Device Information
- Open incognito window

**Search Settings**

- Results per page: 20
- Private results: Do not use
- Region settings: UK

**Search Terms 1**

- **All these words:** community based rehabilitation
- **Any of these words:** mental psychosocial

**Search Terms 2**

- **All these words:** community based inclusive development
- **Any of these words:** mental psychosocial

**Restrictions**

- **Language:** English
- **Safe search:** Off

**Hand searched websites**

*Funders - Date searched: 06/08/20*

- DFAT (Australia)
- DFID DevTracker (UK)
- USAID   (US)
- Disability Rights Fund
- Fondation d’Harcourt
- Tata Trust
- Gulbenkian Foundation
- Open Society Foundation

*Non-governmental organisations - Date searched: 29/07/20*

- BasicNeeds
- CBM
- Handicap International/Humanity and Inclusion
- Making it Work Good practices (Humanity and Inclusion)
- Light for the World
- The Zero Project
- Sangath

*Research organisations - Date searched: 29/07/20*

- CGMH (Centre for Global Mental Health)
- CIRRIE (Centre for International Rehabilitation Research Information & Exchange) – **Excluded - not grey literature**
- FIRAH (Foundation of Applied Disability Research) – **Excluded – not grey literature**
- HDRF (Human Development Research Foundation) – **Excluded – not grey literature**
- ICED (International Centre for Evidence on Disability)
- SCARF

*Advocacy organisations - Date searched: 30/07/20*

- Disabled People’s International
- European Disability Forum
- IDA (International Disability Alliance)
- IIDDC (International Disability and Development Consortium)
- WAPR (World Association for Psychosocial Rehabilitation)
- World Federation for Mental Health
- MindFreedom International
- World Network of Users and Survivors of Psychiatry
- Movement for Global Mental Health
- GPD (Global Partnership on Disability and Development) – **Excluded – couldn’t access**
- Pan-African Network of People with Psychosocial Disabilities - **Excluded – couldn’t access**

*Knowledge brokers - Date searched: 30/07/20*

- MHIN
- MHPSS.net
