# Supplementary material for: Community-based rehabilitation for people with psychosocial disabilities in low- and middle-income countries: a systematic review of the grey literature
Source: Int J Ment Health Syst. 2024 Mar 14;18:13. doi: 10.1186/s13033-024-00630-0 (PMC10941461; doi:10.1186/s13033-024-00630-0)
Supplement: Supplementary file 3 — Additional file 3: Data extraction table; summary of results. [file 13033_2024_630_MOESM3_ESM.docx]

**Additional File 3: Data extraction table; summary of results**

| **No.** | **Programme, Organisation and Funder Names** | **Country (Region), Area** | **Type of disability (Diagnosis), Target group** | **Study design** | **Labelled as CBR?** | **Primary CBR component (minor components)** | **Description of services** | **Outcomes** |
| --- | --- | --- | --- | --- | --- | --- | --- | --- |
| [1](https://unesdoc.unesco.org/ark:/48223/pf0000186588?posInSet=2&queryId=f76e31cb-aa17-4e49-8df6-6b3eae2c5568) | Programme: Training for children with Autism Organisation: The Autism Awareness Care and Training (AACT), [original: https://aactgh.org/course.html], Funder: Unknown | Ghana (Kokomlemle, Accra), Urban | Developmental (Autism), Children | Mixed methods (Routine monitoring and evaluation data reported by programme) | No | Livelihood and Education (Social, Empowerment) | “Originally intended as a resource centre for families, AACT has expanded into an educational site which offers life-skill (such as taking a shower, combing, brushing of teeth, washing, dressing, ironing, etc.) as well as some vocational skills (such as cooking, baking, juicing, salad making, frying, gardening etc.) and academic education as well as speech, art and music therapy. AACT has also partnered with a local pre-school to offer play based inclusive education for some of its youngest students. They also provide opportunities for the children to attend mainstream school to socialise with other people and learn to cope with new environments and situations. AACT also does community awareness campaigns and parent training workshops.” | - “Over 600 children have received training. - The art therapy training has enabled the children to express themselves better and felt understood - These led to the children's social inclusion into some mainstream schools, health care facilities and social gatherings such as churches, markets, shopping malls among others.” |
| [2](https://www.ilo.org/wcmsp5/groups/public/---ed_emp/---ifp_skills/documents/publication/wcms_132675.pdf) | Programme: Shreyas  Organisation: Malankara Catholic Diocese of Bathery, India  Funder: Unknown | India (Sulthan Bathery, Kerala State), Rural | All disabilities, including psychosocial, Adults | Quantitative (Routine monitoring and evaluation data reported by programme) | Yes | Livelihood (Health and Empowerment) | Programme builds capacity of CBR workers to support people with disabilities, helps ensure access to education, benefits and assistive devices, and provides counselling as well as vocational and skills training and livelihoods support. Trainees can access technical, material and financial support services and expert advice on employment and setting up an informal business. Shreyas facilitates financial assistance to income-generating activities via a community bank and self-help groups. The programme also carries out disability awareness-raising interventions. | - Skills training received by 35 people with physical disabilities and an additional 2 with visual, 6 with hearing, 7 with intellectual and 3 with mental health impairments. - Out of the graduates, 57% secured jobs, an additional 26% started their own businesses, and an additional 12% started working on the family business or farm. - Only 5% remain unemployed. Employment data is not disaggregated by type of impairment. |
| [3](https://www.ilo.org/wcmsp5/groups/public/---ed_emp/---ifp_skills/documents/publication/wcms_132675.pdf) | Programme: Samarthya  Organisation: Samuha, Karnataka  Funder: Unknown | India (Koppal and Raichur districts, Karnataka State), Rural and urban slums | All disabilities, including psychosocial, Adults | Quantitative (Routine monitoring and evaluation data reported by programme) | Yes | Livelihood, (Health, Education and Empowerment) | Programme offers therapeutic services, aids and appliances, facilitates village-level self-help groups as well as district- and state-level disabled persons organisations (DPOs), supports school integration, and provides vocational and skills training. Samrthya organises community accommodation, insurance and support for food and transport, and also assists in job searching and business start-up. It collaborates with other organisations to identify suitable employment and income generating opportunities. The programme also carries out disability awareness-raising interventions. | - Skills training received by 170 people with physical disabilities and an additional 11 with visual, 5 with hearing and 1 with mental health impairments, as well as 3 with other disabilities. - Of those trained, 39% have secured jobs, 32% started their own businesses, 16% have started working on the family business or farm, and an additional 0.5% have gone on to further training. - 12% remain unemployed. Employment data is not disaggregated by type of impairment. |
| [4](https://www.ilo.org/wcmsp5/groups/public/---ed_emp/---ifp_skills/documents/publication/wcms_103984.pdf) | Programme: Developing Entrepreneurship among Women with Disabilities (DEWD) project, Funder: Women’s Entrepreneurship Development and Gender Equality (WEDGE). | Ethiopia, Kenya, Tanzania, Uganda and Zambia, Not reported | All disabilities, including psychosocial and mothers of children with intellectual disabilities | Quantitative (Routine monitoring and evaluation) | No | Livelihood and Empowerment | “Supporting women with disabilities and women with disabled dependents in improving their standard of living through training in micro-enterprise skills, vocational skills training as well as access to credit and business development services. A key element of the strategy is the involvement of the disabled persons’ organizations (DPOs) in carrying out project activities. Disabled women entrepreneurs took part in the training programmes run for non-disabled women funded through another project.” | - “443 women with disabilities – including mothers of intellectually-disabled children have received training; 396 received training in basic business skills and 47 in “Improve Your Business” skills - Over 200 women with disabilities and women with disabled dependents received loans to implement their business plans.” |
| [5](https://apps.who.int/iris/bitstream/handle/10665/67266/WHO_MSD_MPS_02.1.pdf?sequence=1) | Programme: Mental Health Policy and Service Development (MPS) project. Organisations: Ministry of Health, Angoda (Teaching) Mental Hospital,  Nivahana Society of Kandy (NGO). Funder: Central Ministry of Health | Sri Lanka (Kandy and Colombo), Urban | Psychosocial, Adults | Mixed methods (Routine monitoring and evaluation) | No | Social (livelihood, Empowerment) | “The programme relocates people recently discharged from mental hospitals in Colombo to the Central Province and provide them with stable housing or integrate them back into family homes. This aims to reduce re-admissions to mental hospitals by establishing effective support systems in the community. The programme also provides vocational training and employment support and raised awareness about mental health in the community.” | - Raised the level of awareness in the community and among policymakers and secured their support. - Decreased the number of readmissions to psychiatric hospitals by approx. 70%. - Establishing forums for carer groups to express their needs and concerns. - Establishing rehabilitation facilities in the community. - Several success stories |
| [6](https://www.ohchr.org/sites/default/files/Documents/HRBodies/CRPD/DGD/2016/Turkey.doc) | Programme: Hope Houses Project Organisation: The Ministry of Family and Social Policies, Funder: Unknown | Turkey, Not reported | Psychosocial and intellectual, Adults | Mixed methods (Routine monitoring and evaluation data reported by programme) | No | Social (livelihood) | “The project provides care services through active participation in society to persons with disabilities who reside in care and rehabilitation centres. The project provides accommodations to a maximum of six disabled people in houses under caretaker supervision and one parent of the disabled individual can be accommodated. The project aims to encourage disabled individuals to adapt easier to daily life by making connections and establishing social relationships with their neighbours while equipping them to live more independently through psycho-social support and support relating to education and employment.” | “The number of such houses reached up to 123 (as of 2017). It has also increased public awareness and helped integrate them in schools and the workforce.” |
| [7](https://www.google.com/url?sa=t&rct=j&q=&esrc=s&source=web&cd=&ved=2ahUKEwjh67zCiL38AhUUacAKHYwCC-AQFnoECBIQAQ&url=https%3A%2F%2Fwww.ohchr.org%2Fsites%2Fdefault%2Ffiles%2FChina.doc&usg=AOvVaw3CuGx3fw-KKw684kjLJoAS) | Programme: Sunshine Home Project, Government funded and implemented | China, Not reported | Psychosocial and intellectual, Adults | Quantitative (Service delivery data reported by project) | Yes | Livelihood (Education, Health) | “The elemental services content include basic living supports and caring (feeding, clearing and toilet, etc.), living skill training, psychological service and behaviour correction, rehabilitation and medical care, social adjustment training, vocational training and vocational rehabilitation. There are day-care community-based facilitates, residential institutes with 24-hour services and home-based visiting services.” | “From 2009-2011, there are 87,000 disabled people using services and 577,000 use home-based services with small subsidy in cash.” |
| [8](http://www.aifoeng.it/archives/project_reports/india_malavalli_annual_report_2016.pdf) | Programme: MALAVALLI CBR, Organisation: Shree Ramana Maharishi Academy for the Blind, Funder: Unknown | India (spread over 1300 villages), Rural | All disabilities, including psychosocial, Adults | Quantitative (Routine monitoring and evaluation data reported by programme) | Yes | Health, Education, Livelihood, Social and Empowerment | “The Health promotion and prevention programme includes health promotion workshops for women, information on assistive devices and access to medication. The education rehabilitation programme includes education guidelines for secondary schools’ students, parental guidelines on the educational schemes that benefit children with disabilities, lifelong learning skills and school awareness programmes. The livelihood support includes vocational support such as self-employment through groups, skills training and vocational placements. The empowerment programmes offer leadership training for self-help groups and disabled people organisations. The programme also provided social rehabilitation which included inclusive sports meets, competitions, celebration of Independence Day and provides them with social security schemes such as insurance.” | - “1606 persons with various disabilities were referred to various rehabilitation centres and hospitals. - 319 people with mental illness and epilepsy received medicine from the project and various donors. - 58 children with special needs admitted in normal school and special school to continue their education. - 135 people with disabilities participated in different skill training and vocational training like computer courses, tailoring etc based on their needs. - 162 people with disabilities got employment in garments, tailoring and various job. - 586 persons received financial aids from various government schemes, subsidiary bank loads and loans from self-help groups. - Legal support and social support provided for 28 person with disabilities.” |
| [9](https://odihpn.org/publication/reflections-on-a-psychosocial-community-support-programme-in-the-west-bank/) | Programme: Multi-Family Approach through Community Based Rehabilitation, Organisation: War Trauma Foundation and the Institute for Community and Public Health (ICPH) at Birzeit University, Funder: Unknown | Palestine (West Bank), Rural | All disabilities including psychosocial, Mothers of children | Qualitative (Routine monitoring and evaluation data reported by programme) | Yes | Education and Empowerment | “The MFA brings together mothers of children with a mental or physical disability to share experiences and learn from each other. One MFA group, for instance, brings together women with autistic children from three West Bank villages, where they receive support from other mothers whose children have the same condition. 5 Sessions involve enjoyment and relaxation for mothers, alongside serious conversations around shared personal, family and community experiences. A group facilitator is present, but since the MFA is a peer-support programme they are there merely to guide the process, not deliver content. The women themselves decide what topics they wish to discuss. There are currently around 40 groups, each consisting of around ten women. Sessions are usually held once a month, or every two months.” | - “Members report that the caring and trusting environment within the groups has enabled them to exchange experiences and talk freely about their children’s problems. - The women also report reduced stigma and feeling less stress. - Their families also notice positive changes at home - Group members are also more confident and are now lobbying at the Ministry of Education for better educational opportunities for their children. |
| [10](http://www.stichting-camelia.nl/RRPAPoct2015.pdf) | Programme: Panti Asih Pakem CBR Program, Organisation: Panti Asih, ZZO Netherlands, Funder: Yayasan Camelia Foundation in the Netherlands. | Indonesia, Not reported | Psychosocial, Young adults | Qualitative (Routine monitoring reports and evaluation, field visits and observations, interviews with program management) | Yes | Livelihood and Social | “An intensive two months training program. The training has three components - (1) healthy lifestyle, (2) skills for productive work, and (3) recreation, arts and sports. Training includes mechanics, women’s skills, handicraft, workshop, agriculture, cooking, protective work, music, sports, and play as well as healthy lifestyle.” | - “Increased ability of young adults with mental disability to meet their own needs through productive activities (goat breeding, or paid work away from home). - Increased awareness amongst families of the importance of skills for young adults with mental disability for their independence, and the families more open attitude towards CBR programs. - Increased awareness of specialized schools of the strategic importance of CBR in helping their students become independent. - Acknowledgement of district government about the importance of CBR program, as testified by the district administrator’s endorsement of the training (by signing the certificate of accomplishment).” |
| [11](http://story.apcdfoundation.org/?q=system/files/TCTP%202019%20Report_RPDF.pdf) | Programme: Third Country Training Programme  Inclusive Development Through Disability-Inclusive Sports, Organisations and Funders: Asia-Pacific Development Centre on Disability, Japan International Cooperation Agency, Thailand International Cooperation Agency of the Ministry of Foreign Affairs of Thailand | Participants from ASEAN countries (Cambodia, Lao PDR, Malaysia, Myanmar, Philippines, Thailand, and Vietnam), Programme located in Thailand, Urban | Developmental (Autism) and Psychosocial, Young athletes and their parents | Mixed methods (Program reports and participant reports) | Yes | Social (Empowerment) | “Training program designed to build capacities of potential athletes with autism and psychosocial disability and their parents as trainers in Track & Field, Cycling and Takkyu Volley towards inclusive participation in any national/ international competitions of persons with autism. The training consisted of games and activities to promote their empowerment through recreation, leisure and sports. Athletes with diverse disabilities from Thailand shared their expertise and technical skills. Workshops took place on social participation and vocational life. Cycling games and competitions took place with medals awarded to boost confidence.” | - “27 participants participated in the TCTP 2019, including 12 athletes with autism and psychosocial disability, and 15 parents/trainers - Participants able to identify their individual and collective barriers and helped them develop skills in sports adaption. These were then positively promoted at the ASEAN Autism Games (AAGs) - Mutual understanding and friendships between participants were developed. - Actual field practice provided an opportunity to challenge commonly held misconceptions about persons with autism and psychosocial disability when it came to demonstrate their capacities.” |
| [12](https://drive.google.com/file/d/15hu3rJlmWUCzGD_ngzph6J8ICfHDRTNh/view?usp=share_link) | Programme: Urban mental health programme, Naya Daur , Day care centre, Sarbari & Marudyan, Reintegration programme, Crust and core, Nayagram. Organisation and Funder: Iswar Sankalpa | India (Kolkata), Urban | Psychosocial, Adults (Homeless) | Mixed methods (Routine monitoring and evaluation data reported by programme) | No | Health, Education, Livelihood, Social and Empowerment | “Iswar Sankalpa aims to provide holistic support to help people with psychosocial disabilities that are homeless or living in under privileged sections of society. The Urban Mental Health Programme aims to prevent homelessness due to mental health conditions by integrating mental health services within the primary urban health care centres and providing early interventions and awareness building. The Community Based Outreach Programme’s key activities include case finding, engagement and assessment, identifying their caregivers and assessing them medically. Treatment and support includes basic needs such as clothes, food, counselling and accommodation in shelters. The organisation created shelters for women and men. Day care centres and rehabilitation activities include self-care and social skills such as gardening, craft, cooking, dance and drama therapies, vocational activities, life skills sessions, functional literacy classes and counselling. The programme then aims to re-integrate the person back into the community and providing literacy training, vocational skills training, supported employment and access to entitlements. Psychoeducation is also provided to the family and after care to including follow up calls, home visits, and hospital linkages. The programme also runs mental health camps in the community to provide immediate care for homeless mentally ill people and encourage the community to take responsibility for their care.” | - “The programme has helped persons to relearn how to look after themselves and be integrated back into the community. - Within the Naya Daur programme, 126 community caregivers take part in the programme resulting in the recovery and improvement of 70% of the clients (measured by the Indian disability evaluation and assessment scale (IDEAS)). - From the Sarbari women’s shelter 70% of the clients recorded an overall improvement in the IDEAS, while 96% of the men in the Marudyan shelter improved. - 110 clients participated in the functional literacy programme, 148 in the vocational programme, and 110 in the supported employment and access to entitlements. - At follow up, the programme reported 207 clients followed up, 29 home visits and 44 clients reunited with their families. |
| [13](https://www.basicneeds.org/self-help-group-stories-promoting-better-mental-health/) | Programme: Basic needs - China Organisation: Basic needs, Funder: Unknown | China, Not reported | Psychosocial, Adults | Qualitative (Observations) | Yes | Empowerment (livelihood) | “A charity bazaar was held by the Liang Fen Zhuang self-help group in the community. The group’s livelihoods activity is planting grapes and the bazaar was an opportunity to sell their produce and raise awareness of mental health by interacting with community members.” | “The bazaar was a great success, many people from the community came to buy the grapes which were sold out. They interacted with the group members and thus reduced stigma in the society. Also, the Yanzhao Metropolis Daily, a local newspaper, reported the story raising more awareness and attention from the media.” |
| [14](https://zeroproject.org/view/project/5f827a3e-9317-eb11-a813-000d3ab9b226) | Programme: Addressing sexual and reproductive rights of people with disabilities, Organisation: Profamilia, ASDOWN Colombia, LICA and PAIIS, Funder: Open society foundation | Colombia, Not reported | Psychosocial and intellectual, Adults | Mixed methods (Monitoring and evaluation data) | No | Empowerment (education) | “First training programme in Colombia on capacity and sexual and reproductive rights for people with intellectual and psychosocial disabilities. The organisation created information cards for judges, health professionals, persons with disabilities, and their families. The “Support in Decision Making in Sexual and Reproductive Health” guide supports people to make decisions about issues like contraception and abortion. This tool was validated with people with psychosocial and intellectual disabilities. It is now being shared with other health providers in Colombia.” | - “Profamilia trained all staff in its 30 sexual health clinics across Colombia. - 209 young people with intellectual disabilities attended training sessions to develop skills to self-advocate and share information through their social networks. - In 2017, the four organizations supported the drafting of the Colombian Ministry of Health’s Resolution 1904, which guarantees the right of persons with disabilities to receive adequate information to exercise their sexual and reproductive rights and prohibits the sterilization of people with disabilities without their consent.” |
| [15](https://zeroproject.org/view/project/46163407-5423-eb11-a813-0022489b3a6d) | Programme: Empowerment through peer-to-peer support Organisation: National Organization of Users and Survivors of Psychiatry (NOUSPR), Funder: Unknown | Rwanda, Not reported | Psychosocial, Adults | Quantitative (Monitoring and evaluation) | No | Livelihood and Empowerment | “Through the collection of personal stories, the project aims at giving a voice to persons with psychosocial disabilities at the community, national, and international level. Peer-to-peer support, economic empowerment, and advocacy lead to their full participation in political and public life and enable self-determination and independent living. Through peer-to-peer support, beneficiaries can better manage and address their individual needs, and families are trained to accommodate persons with disabilities. Income-generating activities increase their standard of living and enable them to contribute financially to their families and communities, which in turn leads to their being recognised as valuable members of society.“ | - “The program currently has some 1,200 members, 79 per cent of whom are persons who have themselves experienced mental health challenges. - 14 self-help groups are actively leading an independent life.” |
| [16](https://www.scarfindia.org/clinical-services/) | Programme: Day care centre Organisation and Funder: Schizophrenia Research Foundation (SCARF India) | India, Not reported | Psychosocial (Schizophrenia), Adults | Qualitative (Case reports) | No | Livelihood (Health) | “This Day care is free of cost, provide free transportation and has cash incentives based on set criteria. Clients are involved in activities ranging from making paper bags, areca plates (pakkumattai plates), printing, gardening, yoga, music etc. This is done with the primary objectives of symptom control, reducing disability, improving functioning and recovery. They also offer services such as Vocational Training (Need-based skill training, Self-employment, Income generation groups) and Vocational rehabilitation (Need-based and driven by choice of the person with mental illness, Graded skill training activities, Various work units, Facilitate for open employment, Ensure sustainability in a job, Networking with employers)” | “Around 60/70 people attend the Day care centre on average. There are many success stories. One being Bano's; She took a tailoring and dress designing course and enrolled in a nearby women’s Polytechnic College for one year. At follow up, Bano has adhered to her treatment, taking medication regularly and also attending scheduled reviews. It has now been five years since her last relapse.” |
| [17](http://www.msctrust.org/rehabilitation-programme/community-based-rehabilitation/district-mental-health-programme-ramnad/) | Programme: Ramnad District Mental Health Programme Organisation: MSChellamithu Trust and Research Foundation, Funder: Unknown | India (Ramanathapuram), Rural | Psychosocial, Adults | Mixed methods (Monitoring and evaluation data) | Yes | Livelihood, Health (Social, education, and Empowerment) | “The programme initially sensitized the entire district officers and officials educating and training them on basic psychiatry/ mental health. Also, an awareness programme was conducted all around the district to educate and reduce stigma. This was done through 125 street plays around the district. Also 12 Community mental health camps were organised providing health care, treatment programmes and entitlements (such as disability certificate, free bus pass, free food grains) to people with mental disabilities that have been chained. 11 rehabilitation centres then provided people with a range of activities including vocational training and employment placements to help recover and reintegrate them into the community. Other activities in training centres included occupational training, physiotherapy, play activities, family education, life skills training and training on work behaviour and under personal skills. Those who attend the training sessions also receive extended benefits: Free bus pass, Rs.200/- per month as incentives, Free medical care & medicine and Free food. The persons families are also involved and educated on mental health. Self-help groups have also been formed at district level.” | - “People who have attended the training centres were given work opportunities and generated continuous income. They were regularly followed up. - The recovered / trained persons are motivated to start their own enterprises with the financial assistance of District Administration which in turn helped them to enhance their Quality of Life and their social dignity. - Family members are also now educated to manage disabled members of the family and are also involved in the promotion and sales of the goods manufactured by the mentally disabled persons. - 180 self-help groups have been opened and most have opened their own bank account.” |
| [18](http://www.msctrust.org/rehabilitation-programme/community-based-rehabilitation/community-mental-health-camp-sivakasi/) | Programme: Rural Community Mental Health Camp, Organisation: Sri Sathya Sai Samithi Volunteers of Sivakasi, M.S.Chellamuthu Trust and Research Foundation, and Mahatma Montessori Higher Secondary School, Madurai, Funder: Unknown | India (Sivakasi), Rural | Psychosocial, Adults (Homeless) | Mixed methods (Routine monitoring and evaluation data) | Yes | Livelihood and Health (Empowerment) | “The community mental health camp is run every 3rd Sundays by the volunteers. They are given vocational training programmes, like goat rearing, mushroom cultivation, soap making, etc. Awareness programme on mental health and mental disabilities are also frequently conducted for Sai volunteers and for the general public. The recovered people are then placed in different work industries depending upon their capability. Free food is also made available to those who attend camp.” | - “1115 persons with mental disabilities were identified from the streets, assessed, treated and rehabilitated with the resources in the community. - 65% of those who attended camps are employed and successfully living a quality life. - 23 self-help groups have been formed and have launched community enterprises such as a baker, department store, laundry services and a weighing machine. - The increased awareness is also reducing stigma in the community.” |
| [19](https://www.mhinnovation.net/innovations/mental-health-rehabilitation-homeless-populations-nigeria?qt-content_innovation=0#qt-content_innovation) | Programme: Amaudo Itumbauzo  Organisation: Methodist Church, Nigeria Funder: Abia State Government, Nigeria; Amaudo, UK; Methodist Church, Nigeria | Nigeria (Southeast Region: Originally Abia State, scaled up to also cover Anambra, Ebonyi and Imo States), Rural | Psychosocial, Adults (Homeless) | Quantitative (Routine monitoring and evaluation data compiled externally by external evaluator) | Yes | Livelihood and Health (Social and Empowerment) | “Programme integrates medical, social, occupational and psychological services, targeting both the user and family for purposes of rehabilitation and reintegration. Operates a residential rehabilitation centre and a network of 73 community mental health clinics where users can access decentralised mental health care following reintegration into the community. All clients receive vocational training prior to discharge and are then also supported to pursue their chosen vocation in their home setting. Programme also carries out mental health awareness-raising interventions to combat stigma and improve detection, particularly in rural communities, and supports self-help groups.” | - “930 people with mental illness who were destitute have been rehabilitated, reunited with their families and reintegrated into their communities. - Over 3000 mentally ill clients visit the community mental health clinics monthly for treatment thereby bridging the gap created by government psychiatric hospitals which are located in big cities - 9 Service User Groups or Self-Help Groups have been formed - Over 3000 student nurses have participated in community mental health care training and experience from Amaudo.” |
| [20](https://www.mhinnovation.net/innovations/self-help-groups-mental-health?qt-content_innovation=0#qt-content_innovation) | Programme: The Presbyterian Community Based Rehabilitation (PCBR) Organisations: Presbyterian Community Based Rehabilitation (Ghana), CBM and Basic Needs. Funders: CBM, BasicNeeds and Presbyterian Church of Ghana | Ghana (Sandema, the upper east region of northern Ghana), Rural | Psychosocial, Adults | Qualitative study (Six focus groups and four interviews) | Yes | Empowerment (livelihood) | “The programme establishes self-help groups with the aim of providing mutual support and enabling people with psychosocial disabilities and their families to re-enter the community, both socially and economically. The groups promote self-help, self-reliance and independent decision-making. The activities aim at strengthening the entrepreneurial capacity of the individual members supporting their independent livelihood and other workshops focus on improvement of daily living skills. Persons with psychosocial disabilities have actively participated in the formation of the SHGs, organization structure and decision-making process. They themselves are responsible for defining the agenda of the meetings, thematic priorities and advocacy initiatives.” | - “23 SHGs established in the upper east region of Ghana, each with up to 100 members. - The SHG members report that they are more aware of their own rights and that the discrimination by family members and the community reduced as they gained financial independence. - They feel more confident to challenge discrimination, and to take initiatives that will improve their lives. - 15 advocacy campaigns led by SHG members resulted in an observed decrease in the level of discrimination against people with psychosocial disabilities in Northern Ghana” |
| [21](https://www.mhinnovation.net/innovations/mhgap-implementation-edawu-nigeria?qt-content_innovation=2#qt-content_innovation) | Programme: Edawu Community Mental Health Care Project Organisation: Leicestershire Partnership NHS Trust, Comprehensive Community Mental Health Programme, Otukpo Benue State Nigeria Funders: Wesley Guild ACCEPT, Methodist Church Nigeria | Nigeria (Edayu, Benue State), Rural | Psychosocial, Adults (Homeless) | Mixed methods (Programme evaluated the training through service statistics and questionnaires to measure individuals’ subjective perspective of improvement) | No | Health (empowerment) | “The project oversees a community psychiatric programme which includes clinical practice and focus on providing in-patient care and rehabilitation for homeless people with follow-up after discharge. The programme also devised a strategy for early identification of mental illness by delivering mhGAP training through staff workshops, role playing activities and lectures. They also developed a community mental health awareness programme.” | - “The centre has rehabilitated over 10,000 people suffering from mental illness - Increased the awareness of mental health in the local area. - The number of homeless people roaming the local market has reduced and is now a rarer sight - Approximately 20 people who were previously homeless were trained in various vocational and empowerment skills and now lead productive lives.” |
| [22](https://www.mhinnovation.net/innovations/protected-home-hogar-protegido-carabayllo?qt-content_innovation=0#qt-content_innovation) | Programme: Carabayllo Protected Home, Organisation: Partners In Health (PIH) Peru,Ministry of Health and the Municipality of Carabayllo Funder: Partners in Health | Peru (Lima, District Carabayllo), Urban | Psychosocial, Adults (Women) | Mixed methods (Monitoring and evaluation activities included planned monthly visits to the Protected Home for direct observation, incident, visit and case records, income reports, economic expenses and inventory tracking) | No | Social (Health, Livelihood) | “This programme provides housing and rehabilitation care for women with chronic mental illnesses in the event of familial and social abandonment. The programme trained community health agents, called "Cuidadoras (caretakers)" to care for the residents. The training included modules on recognizing mental health disorders, behaviour management, delivering better treatment and building social skills. Caretakers are responsible for conducting self-care workshops, individual and group therapies for the residents of the Protected Home. Some activities include: Building self-care skills Developing domestic autonomy Social skills and community integration Daily routine structure Health and psychoeducation Occupational reintegration” | - “The programme has trained 10 community agents and houses 6 female residents. - There has been a reduction in levels of social exclusion, disability, clinical dysfunction and costs for people living in a sheltered home compared to a similar institutionalized population. - All of the residents are actively participating in the community; 3 have enrolled to complete their state education and 4 of the residents have engaged in economic activities.” |
| [23](https://www.mhinnovation.net/innovations/community-based-rehabilitation-severe-disorders?qt-content_innovation=2#qt-content_innovation) | Programme: Ashagram CBR Programme  Organisation: Ashagram, India Funder: ActionAid India | India (Barwani District, Madhya Pradesh State), Rural | Psychosocial (Schizophrenia), Adults | Quantitative (Longitudinal study comparing outcomes of community-based rehabilitation model with outpatient care) | Yes | Health (Social, Empowerment) | “The Ashagram centre already operates a mental health clinic. The CBR programme further trains local community members as CBR workers to provide comprehensive home-based services, e.g. detection, psychoeducation, follow-up and monitoring, planning rehabilitation interventions and liaising with providers. Most villages formed self-help groups for social and economic reintegration. Programme also carries out mental health awareness-raising interventions to combat stigma and improve detection. “ | - “Greater change in Disability Assessment Scale (DAS) and Positive and Negative Syndrome Scale (PANSS) in CBR group compared to outpatient group. - More than 7,000 persons with mental disorders have been assessed and provided with outpatient treatment. - 600+ persons have been provided with CBR services and 70+ self-help groups comprising of people with severe mental disorders, their family members and others were formed to improve social inclusion and access to micro-credit in villages” |
