# Supplementary material for: Community-based rehabilitation for people with psychosocial disabilities in low- and middle-income countries: a systematic review of the grey literature
Source: Int J Ment Health Syst. 2024 Mar 14;18:13. doi: 10.1186/s13033-024-00630-0 (PMC10941461; doi:10.1186/s13033-024-00630-0)
Supplement: Supplementary file 4 — Additional file 4: Quality assessment scoring and Inter-rater score. [file 13033_2024_630_MOESM4_ESM.docx]

**Additional File 4: Quality assessment scoring and Inter-rater score**

| **No.** | **Programme Names** | **Type of evaluation data** | **Authority** | **Accuracy** | **Coverage** | **Objectivity** | **Date** | **Significance** | **Quality score** | **Inter- rater agreement score (%)** |  |
| --- | --- | --- | --- | --- | --- | --- | --- | --- | --- | --- | --- |
| [1](https://unesdoc.unesco.org/ark:/48223/pf0000186588?posInSet=2&queryId=f76e31cb-aa17-4e49-8df6-6b3eae2c5568) | The Autism Awareness Care and Training | Case study in external report | YES | PARTLY | YES | YES | YES | YES | 11 | 100% |  |
|  |  |  |  |  |  |  |  |  |  |  |  |
|  |  |  |  |  |  |  |  |  |  |  |  |
|  |  |  |  |  |  |  |  |  |  |  |  |
|  |  |  |  |  |  |  |  |  |  |  |  |
| [2](https://www.ilo.org/wcmsp5/groups/public/---ed_emp/---ifp_skills/documents/publication/wcms_132675.pdf) | Shreyas | Case study in good practice guide | YES | YES | YES | YES | YES | YES | 12 | 96% |  |
| [3](https://www.ilo.org/wcmsp5/groups/public/---ed_emp/---ifp_skills/documents/publication/wcms_132675.pdf) | Samuha Samarthya | Case study in good practice guide | YES | YES | YES | YES | YES | YES | 12 | 96% |  |
|  |  |  |  |  |  |  |  |  |  |  |  |
|  |  |  |  |  |  |  |  |  |  |  |  |
| [4](https://www.ilo.org/wcmsp5/groups/public/---ed_emp/---ifp_skills/documents/publication/wcms_103984.pdf) | Developing Entrepreneurship among Women with Disabilities | Case study in literature review | YES | YES | YES | YES | YES | YES | 12 | 100% |  |
|  |  |  |  |  |  |  |  |  |  |  |  |
| [5](https://apps.who.int/iris/bitstream/handle/10665/67266/WHO_MSD_MPS_02.1.pdf?sequence=1&isAllowed=y) | Mental Health Policy and Service Development | External policy and project report | YES | YES | YES | YES | YES | YES | 12 | 100% |  |
|  |  |  |  |  |  |  |  |  |  |  |  |
|  |  |  |  |  |  |  |  |  |  |  |  |
| [6](https://www.ohchr.org/_layouts/15/WopiFrame.aspx?sourcedoc=/Documents/HRBodies/CRPD/DGD/2016/Turkey.doc&action=default&DefaultItemOpen=1) | Hope Houses | Country policy report | PARTLY | PARTLY | NO | NO | YES | UNCLEAR | 4 | 96% |  |
| [7](https://www.ohchr.org/_layouts/15/WopiFrame.aspx?sourcedoc=/Documents/Issues/Disability/LiveIndependently/China.doc&action=default&DefaultItemOpen=1) | Sunshine Home | Country policy report | YES | PARTLY | NO | PARTLY | YES | PARTLY | 7 | 100% |  |
| [8](http://www.aifoeng.it/archives/project_reports/india_malavalli_annual_report_2016.pdf) | MALAVALLI CBR | Internal project report | NO | PARTLY | NO | NO | YES | YES | 5 | 92% |  |
|  |  |  |  |  |  |  |  |  |  |  |  |
|  |  |  |  |  |  |  |  |  |  |  |  |
|  |  |  |  |  |  |  |  |  |  |  |  |
|  |  |  |  |  |  |  |  |  |  |  |  |
|  |  |  |  |  |  |  |  |  |  |  |  |
|  |  |  |  |  |  |  |  |  |  |  |  |
| [9](https://odihpn.org/wp-content/uploads/2018/06/HE-72-web.pdf) | Multi-Family Approach | Case study in article | YES | YES | YES | YES | YES | YES | 12 | 100% |  |
|  |  |  |  |  |  |  |  |  |  |  |  |
| [10](http://www.stichting-camelia.nl/RRPAPoct2015.pdf) | Panti Asih Pakem CBR Program | Internal project report | NO | PARTLY | YES | YES | YES | YES | 9 | 100% |  |
|  |  |  |  |  |  |  |  |  |  |  |  |
|  |  |  |  |  |  |  |  |  |  |  |  |
|  |  |  |  |  |  |  |  |  |  |  |  |
| [11](http://story.apcdfoundation.org/?q=system/files/TCTP%202019%20Report_RPDF.pdf) | Third Country Training Programme Inclusive Development | Internal annual report | PARTLY | YES | NO | PARTLY | PARTLY | YES | 7 | 100% |  |
|  |  |  |  |  |  |  |  |  |  |  |  |
|  |  |  |  |  |  |  |  |  |  |  |  |
|  |  |  |  |  |  |  |  |  |  |  |  |
| [12](https://drive.google.com/drive/folders/13x-MoQRZ5KHA9yf75deSi7tAUKVYU9WH) | Urban mental health programme, Iswar Sankalpa | Internal annual report | PARTLY | YES | YES | PARTLY | PARTLY | YES | 9 | 92% |  |
|  |  |  |  |  |  |  |  |  |  |  |  |
|  |  |  |  |  |  |  |  |  |  |  |  |
|  |  |  |  |  |  |  |  |  |  |  |  |
|  |  |  |  |  |  |  |  |  |  |  |  |
|  |  |  |  |  |  |  |  |  |  |  |  |
|  |  |  |  |  |  |  |  |  |  |  |  |
|  |  |  |  |  |  |  |  |  |  |  |  |
|  |  |  |  |  |  |  |  |  |  |  |  |
| [13](https://www.basicneeds.org/self-help-group-stories-promoting-better-mental-health/) | Basic needs - China | Website not found | | | | | | | | |  |
| [14](https://zeroproject.org/practice/pra191025col-factsheet/) | Addressing sexual and reproductive rights of people with disabilities | External website report | PARTLY | PARTLY | YES | YES | PARTLY | YES | 9 | 100% |  |
|  |  |  |  |  |  |  |  |  |  |  |  |
|  |  |  |  |  |  |  |  |  |  |  |  |
| [15](https://zeroproject.org/practice/national-organization-of-users-and-survivors-of-psychiatry-nouspr-rwanda/) | Empowerment through peer-to-peer support | External website report | PARTLY | NO | NO | NO | NO | NO | 1 | 100% |  |
|  |  |  |  |  |  |  |  |  |  |  |  |
| [16](https://www.scarfindia.org/clinical-services/) | Day care centre (SCARF India) | Internal website | PARTLY | PARTLY | YES | YES | NO | PARTLY | 7 | 100% |  |
|  |  |  |  |  |  |  |  |  |  |  |  |
| [17](http://www.msctrust.org/rehabilitation-programme/community-based-rehabilitation/district-mental-health-programme-ramnad/) | Ramnad District Mental Health Programme | Internal project report | NO | NO | NO | PARTLY | NO | PARTLY | 2 | 100% |  |
|  |  |  |  |  |  |  |  |  |  |  |  |
|  |  |  |  |  |  |  |  |  |  |  |  |
|  |  |  |  |  |  |  |  |  |  |  |  |
| [18](http://www.msctrust.org/rehabilitation-programme/community-based-rehabilitation/community-mental-health-camp-sivakasi/) | Rural Community Mental Health Camp | Internal project report | NO |  |  |  |  |  |  |  |  |
|  |  |  |  | NO | NO | PARTLY | PARTLY | PARTLY | 3 | 100% |  |
|  |  |  |  |  |  |  |  |  |  |  |  |
|  |  |  |  |  |  |  |  |  |  |  |  |
| [19](https://www.mhinnovation.net/innovations/mental-health-rehabilitation-homeless-populations-nigeria?qt-content_innovation=0#qt-content_innovation) | Amaudo Itumbauzo | External website report | PARTLY | PARTLY | YES | YES | NO | PARTLY | 7 | 100% |  |
|  |  |  |  |  |  |  |  |  |  |  |  |
|  |  |  |  |  |  |  |  |  |  |  |  |
|  |  |  |  |  |  |  |  |  |  |  |  |
|  |  |  |  |  |  |  |  |  |  |  |  |
| [20](https://www.mhinnovation.net/innovations/self-help-groups-mental-health?qt-content_innovation=0#qt-content_innovation) | The Presbyterian Community Based Rehabilitation | External website report | PARTLY | PARTLY | YES | YES | NO | PARTLY | 7 | 100% |  |
|  |  |  |  |  |  |  |  |  |  |  |  |
|  |  |  |  |  |  |  |  |  |  |  |  |
| [21](https://www.mhinnovation.net/innovations/mhgap-implementation-edawu-nigeria?qt-content_innovation=2#qt-content_innovation) | Edawu Community Mental Health Care Project | External website report | PARTLY | PARTLY | YES | YES | YES | PARTLY | 9 | 100% |  |
| [22](https://www.mhinnovation.net/innovations/protected-home-hogar-protegido-carabayllo?qt-content_innovation=0#qt-content_innovation) | Carabayllo Protected Home | External website report | PARTLY | PARTLY | YES | YES | NO | PARTLY | 7 | 100% |  |
|  |  |  |  |  |  |  |  |  |  |  |  |
|  |  |  |  |  |  |  |  |  |  |  |  |
|  |  |  |  |  |  |  |  |  |  |  |  |
|  |  |  |  |  |  |  |  |  |  |  |  |
|  |  |  |  |  |  |  |  |  |  |  |  |
|  |  |  |  |  |  |  |  |  |  |  |  |
| [23](https://www.mhinnovation.net/innovations/community-based-rehabilitation-severe-disorders?qt-content_innovation=2#qt-content_innovation) | Ashagram CBR Programme | External website report | YES | YES | YES | YES | YES | PARTLY | 11 | 100% |  |
|  |  |  |  |  |  |  |  |  |  |  |  |
|  |  |  |  |  |  |  |  |  |  |  |  |
