# Supplementary material for: Community-based rehabilitation for people with psychosocial disabilities in low- and middle-income countries: a systematic review of the grey literature
Source: Int J Ment Health Syst. 2024 Mar 14;18:13. doi: 10.1186/s13033-024-00630-0 (PMC10941461; doi:10.1186/s13033-024-00630-0)
Supplement: Supplementary file 5 — Additional file 5: Additional extraction: Reported challenges to implementation. [file 13033_2024_630_MOESM5_ESM.docx]

**Additional File 5: Additional extraction: Reported challenges to implementation**

| **Themes** | **Programme, Location** | **Direct quotes** |
| --- | --- | --- |
| Stigma | Shreyas, Sulthan Bathery, India  Samuha Samarthya, Karnataka, India | “Negative community and family attitudes towards disabled people in the workforce is also a major barrier.”  “Cultural norms and prejudice discourage women graduates from moving from their home villages to take up opportunities in the city.” |
|  | Developing Entrepreneurship among Women with Disabilities, Ethiopia, Kenya, Tanzania, Uganda and Zambia | “Prevailing attitudes about certain types of disability, has resulted in a clear bias towards those, such as ambulant women with mobility impairments, who need the least or most easily provided reasonable accommodations, whereas those who may have greater support needs, such as women with mental health difficulties, have largely not been included to date.” |
|  | Multi-Family Approach, Palestine | “Facilitators occasionally need to explain the purpose of the group meetings to potential participants, and how they can benefit from them. Women may have negative attitudes towards receiving mental health support because of the stigma that surrounds mental health issues in the West Bank.” |
|  | Amaudo Itumbauzo, Nigeria | “Poor knowledge of mental health issues in the community, therefore high levels of stigmatization and discrimination of people with mental health under rehabilitation by family and society. These result in poor care at home and high risk of relapse.”  “Lack of human right based mental health law in Nigeria. This increases the chances of human rights abuses against people with mental illness and makes advocacy difficult” |
|  | The Presbyterian Community Based Rehabilitation, Ghana | “Some clients have been discouraged by their family members from joining the groups, either because they do not want them to become independent or to associate with other people with psychosocial disabilities. The attitudes of such family members have prevented the groups from growing as much as they could have grown.” |
| Limited/lack of resources | Mental Health Policy and Service Development, Sri Lanka | “The project had to adapt their approaches in the two different districts due to shortages in human resources.” |
|  | Multi-Family Approach, Palestine | “The CBR has struggled to provide adequate support to very vulnerable groups, such as mothers with severely disabled children who need continuous care. This is a difficult group to cater to because the children need to be looked after while the mothers are attending the MFA group. In some groups youth volunteers are available to do this, but these children typically need special care, which the volunteers are not able to provide without specific training.” |
|  | Panti Asih Pakem CBR, Indonesia | “Challenges in sustainability - People demanding free in-house rehabilitation services and so campaigns had no success in fundraising. Panti Asih has experienced human resources to care for people with mental disability but not for program development and management. Hence for sustainability, the two challenges necessitate consistent commitment of internal and external stakeholders.” |
|  | The Presbyterian Community Based Rehabilitation, Ghana | “The financial constraint of a small budget is an ongoing challenge of the innovation” |
|  | Edawu Community Mental Health Care Project, Nigeria | “Successful implementation of mhGAP at Edawu following the training will require regular supervision and appropriate IT support is crucial to support this.  The remote location of the project while increasing coverage of mental health services in rural areas of Nigeria also makes it difficult to attract qualified staff who may be used to urban lifestyles. More experienced staff are likely to leave for better opportunities in urban areas leading to high rates of staff turnover.  Limited resources for mental health treatment provision (e.g. medicines and staff) in remote areas like Edawu, can create challenges in implementing mhGAP treatment plans in the long run” |
|  | Carabayllo Protected Home, Peru | “Constant supervision required by the caregivers managing resident behaviors given the diversity of mental health problems in the Protected Home. –  Difficulty finding appropriate community health workers for the recruitment of caregivers. Caregivers needed to possess strong interpersonal qualities that are not commonly found in this cadre of health workers, but which are needed for the adequate management of any problems that may arise in the Protected Home” |
|  | Ashagram CBR, India | “Severe lack of financial and human resources.” |
| Transport | Shreyas, Sulthan Bathery, India | “As this organization is based in a rural area, transport is a significant barrier.” |
|  | Mental Health Policy and Service Development, Sri Lanka | “Transport was also a barrier, thus the project established transport services to encourage as many people to attend meetings and training.” |
|  | Multi-Family Approach, Palestine | “Programme chose to work with women only as fathers often work from long distances and was difficult for them to attend sessions, and the women were expected to share their experience and learning from the support groups at home.” |
|  | Ashagram CBR, India | “Poor accessibility to many sections of the catchment area” |
|  | Amaudo Itumbauzo, Nigeria | “Change in government policy that affect social services like the ban of the use of motorcycles in cities in Abia State had made it challenging for service users to visit the community mental health clinics” |
| High levels of poverty | The Presbyterian Community Based Rehabilitation, Ghana | “Membership within the SHGs requires a small membership fee. Despite keeping the member contributions to a minimum, the high level of poverty in the area restricts certain clients from joining the groups.” |
|  | Ashagram CBR, India | “Challenging social demographics of the region: High rates of poverty (more than 80% of the catchment area population households were below the designated poverty line)- Large proportion of people from very disadvantaged social groups (like indigenous persons)” |
| Logistical challenges | Amaudo Itumbauzo, Nigeria | Poor provision of social infrastructure like roads and electricity by the government leads to logistical difficulties for the programme, adding burden and costs to travel  “Insecurity in Nigeria like the increased rates of kidnapping in South East and Boko Haram and the herdsmen/farmers clashes in the North made it difficult for foreign partners to visit Nigeria or travel freely” |
|  | Mental Health Policy and Service Development, Sri Lanka | “The lack of infrastructure for follow up and family support in the districts led to frequent readmissions and family rejection. Thus, the project had to restructure services” |
|  | Ashagram CBR, India | “Poor state of the public health system and absence of any mental health services locally prior to development of Ashagram outpatient clinic” |
|  | Edawu Community Mental Health Care Project, Nigeria | “The Edawu project only has one laptop with access to an internet connection through a cellular sim card. Mobile internet in rural areas like Edawu can often be fragile and unpredictable.” |
| Stakeholder engagement | Amaudo Itumbauzo, Nigeria | “Difficulty engaging with governance for advocacy purposes and persuading policy makers to design or implement effective policies for mental health care” |
| Unrealistic expectations | Multi-Family Approach, Palestine | “They may also have unrealistic expectations about the impact of MFA on their lives, and some may join a group in search of support beyond mental health, such as income-generating initiatives or medicine for their disabled children.” |
|  | The Presbyterian Community Based Rehabilitation, Ghana | “New members sometimes have high expectations that being a member will bring financial gain to themselves. These expectations have to be managed carefully in order to maintain commitment from group members.” |
| Appropriateness of training content and materials | Samuha Samarthya, Karnataka, India | “Limited English language proficiency can present difficulties for trainees in some vocational streams, particularly the computer course.” |
